# Loop closure grasping: Topological transformations enable strong, gentle, and versatile grasps

**DOI:** 10.1126/sciadv.ady9581

**Published:** 2025-12-10

**Authors:** Kentaro Barhydt, O. Godson Osele, Sreela Kodali, Cosima du Pasquier, Chase M. Hartquist, H. Harry Asada, Allison M. Okamura

**Affiliations:** ^1^ Department of Mechanical Engineering, Massachusetts Institute of Technology, Cambridge, MA 02139, USA.; ^2^ Department of Mechanical Engineering, Stanford University, Stanford, CA 94305, USA.; ^3^ Department of Mechanical and Aerospace Engineering, University of Florida, Gainesville, FL 32611, USA.

## Abstract

Grasping mechanisms must both create and subsequently hold grasps that permit safe and effective object manipulation. Existing mechanisms address the different functional requirements of grasp creation and grasp holding using a single morphology but have yet to achieve the simultaneous strength, gentleness, and versatility needed for many applications. We present “loop closure grasping,” a class of robotic grasping that addresses these different functional requirements through topological transformations between open-loop and closed-loop morphologies. We formalize these morphologies for grasping, formulate the loop closure grasping method, and present principles and a design architecture that we implement using soft growing inflated beams, winches, and clamps. The mechanisms’ initial open-loop topology enables versatile grasp creation via unencumbered tip movement, and closing the loop enables strong and gentle holding with effectively infinite bending compliance. Loop closure grasping circumvents the tradeoffs of single-morphology designs, enabling grasps involving historically challenging objects, environments, and configurations.

## Teaser

Topological loop closure simultaneously enables versatile grasp creation and strong yet gentle object holding.

## INTRODUCTION

A successful grasp involves (i) creating a stable grasp and (ii) subsequently holding that grasp in a manner that permits safe and effective object manipulation. These two stages of grasping have different functional and performance requirements. For the grasp creation stage, the grasping mechanism must articulate itself around the object to assume a desired, stable grasping configuration. Different objects and environments require different configurations, so the grasping mechanism must be versatile enough to achieve a wide variety of configurations. Improving versatility has long been a major motivation in grasping mechanism design research, especially soft mechanisms that can adapt to a wide variety of complex and unknown shapes (*1*–*5*). For the holding stage, the grasping mechanism must continuously resist destabilizing forces (e.g., gravitational, inertial, and disturbance) during object manipulation to maintain the stable grasp without applying harmful contact pressures. This stage can be especially challenging for heavy yet fragile objects, which weigh much more than their allowable interaction forces, because of the conflict between the need for high forces to lift them and low force concentrations to avoid damage.

These different functional requirements for the different stages of grasping lead to distinct, and potentially conflicting, design needs. Existing grasping mechanisms use a single static morphology (*1*–*6*) and have yet to achieve the simultaneously strong, gentle, and versatile performance needed for many high-impact applications involving heavy yet fragile objects. While previous designs have advantages for some of these performance aspects, none have been able to excel in all of them. For example, safely securing and lifting humans require bulky manual tools and handler training due to their weight, fragility, pliability, articulation, and wide variance in shape and size. Handling humans is central to many critical applications such as elder care, care of people with physical disabilities, emergency medical response, search and rescue, physical rehabilitation, occupational therapy, and ergonomic support for manual labor (*7*–*14*). Handling heavy yet fragile objects is also critical for many industry applications, such as agricultural harvesting, handling livestock, aircraft and ship manufacturing, civil and architectural engineering, construction, salvage recovery/excavation, and machinery installation (*15*–*21*). However, these applications also require manual tools and trained human operators to harness and handle their respective payloads. Grippers designed to bear high loads exist, but they can only grasp durable objects with no risk of damage and cannot handle a wide variety of objects, grasping configurations, and environments [e.g., heavy manufacturing robots (*22*, *23*) and previous patient lifting robots (*24*, *25*)].

**Fig. 1.**
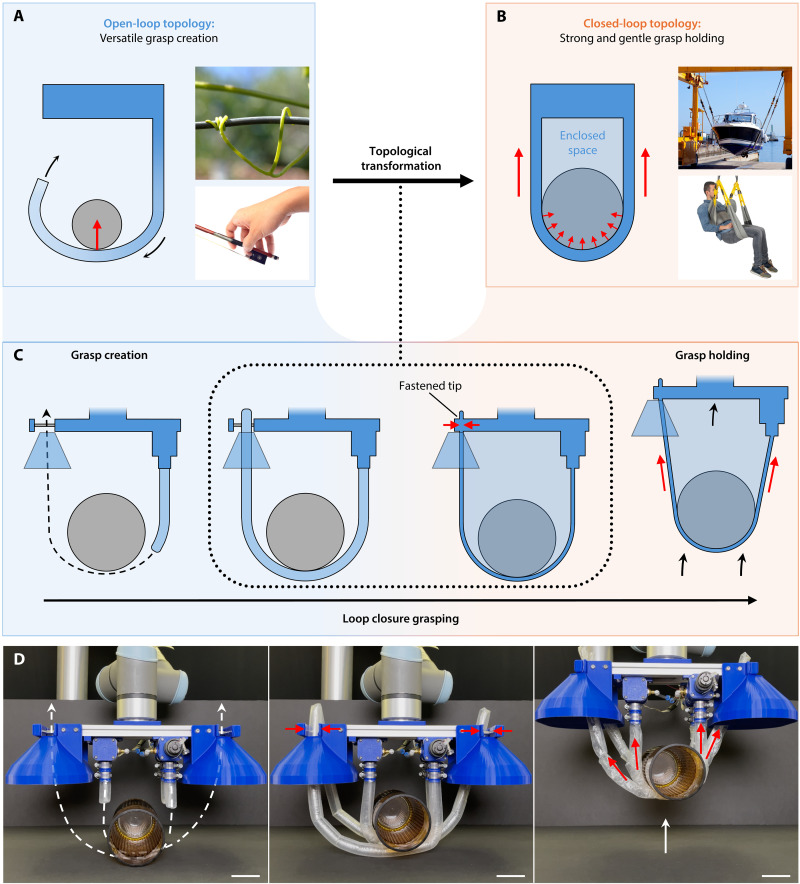
An overview of loop closure grasping. The grasping process can be decomposed into two stages, grasp creation and grasp holding, connected by topological transformation. (**A**) Grasping mechanisms with open-loop topologies are advantageous for versatile grasp creation, as illustrated by biological open-loop grasping mechanisms. (**B**) Grasping mechanisms with closed-loop topologies are advantageous for strong and gentle grasp holding, as illustrated by examples of closed-loop grasping mechanisms. (**C**) Loop closure grasping process. Topological transformations enable mechanisms to transition their morphology from open loop to closed loop. By transforming its topology from open loop to closed loop between the grasp creation and holding stages, a grasping mechanism can leverage the advantages of the right topology at the right stage. (**D**) A loop closure grasping system prototype grasping and lifting a glass vase. Scale bars, 5 cm. Image credit: L. C. Pan, Alamy Ltd. and Oboltus, iStockPhoto LP (A) and TONO BALAGUER, INMAGINE LAB PTE LTD. and not applicable, Surehands Lift and Care Systems (B). Note that we have the licenses and/or permission to use these images and are entitled to use these materials by their associated owners.

We present a method of grasping that transforms the topology of the grasping mechanism’s morphology between open- and closed-loop to enable the creation and holding of simultaneously strong, gentle, and versatile grasps (Fig. 1). Our holistic method, which we call “loop closure grasping,” is centered specifically around this transformation in topology because of the fundamentally distinct and converse advantages of open- and closed-loop topologies for the creation and holding stages of the grasping process, respectively. By strategically enabling and using transformations in the grasping mechanism’s morphology between both topologies, their advantages can be leveraged for the appropriate stage of grasping, and their disadvantages for the other stage can be bypassed. Thus, the tradeoffs of previous single-morphology designs can be circumvented. We additionally elucidate these advantages and disadvantages of open- and closed-loop morphologies to inform the loop closure grasping method and present a holistic grasping system design architecture and principles for enabling and leveraging this method. Prior examples of dynamically changing robot morphologies include reconfigurable robots and robots that adapt their morphology to their environment (*26*–*29*). Unlike these previous works, loop closure grasping uses morphological changes for grasping.

Open-loop mechanisms are advantageous for creating versatile grasps, as their tips can be moved freely around the object and environment to navigate the mechanism into a desired grasping configuration. Nearly all past grasping mechanisms have followed this open-loop paradigm, which has resulted in advances in versatile grasp creation (*1*–*6*, *30*–*38*).

However, current open-loop grasping mechanisms still cannot effectively hold heavy yet fragile objects on the order of heavy industry payloads, humans, etc. (*1*–*4*, *6*, *25*, *30*–*32*, *39*, *40*). Holding strong grasps with open-loop mechanisms requires substantial flexural rigidity to resist destabilizing forces (see Results for the generalized principle), limiting the compliance of the grasp. Compliance enables gentle interactions by passively distributing applied forces [as is well established in soft robotics literature (*2*, *3*, *41*–*44*)]. Thus, a tradeoff exists between how strong and gentle the grasp can be.

Conversely, closed-loop mechanisms are advantageous for holding strong and gentle grasps but are limited in creating versatile grasps. Flexible closed-loop mechanisms that hold objects in a cradled suspended state can bear high loads while passively distributing them over a large contact area. These mechanisms have been used for millennia (e.g., ropes and slings) (*45*–*48*) to safely hold heavy yet fragile objects (*17*–*21*, *49*). Their load capacity depends only on their tensile strength, not their flexural rigidity (see Results for generalized principle). Thus, they can hold objects with theoretically “infinite bending compliance” (zero flexural rigidity), taking one of the strongest motivations for soft robotics [compliance (*41*–*44*)] to one of its theoretical extremes.

However, permanently closed-loop grasping mechanisms are limiting for versatile grasp creation. In two dimensions, an object cannot physically enter a closed loop. In three dimensions, the entire loop must maneuver around the object at once due to lacking a free tip (unlike open-loop continuum and hyperarticulated robots that only need to navigate their tips). Thus, available paths through which it can navigate to achieve stable grasps are limited. Most current uses of closed-loop mechanisms to hold objects require external work to securely harness them (*17*–*21*, *49*) [e.g., a human manually wrapping a sling around a heavy industry payload for lifting (*7*)], and the few that can autonomously create grasps are limited in their versatility to specific objects in controlled environments (*50*, *51*).

By enabling the mechanism to dynamically transform between open- and closed-loop topologies, loop closure grasping can create and hold grasps that leverage the advantages of the right topology at the right stage. This eliminates the tradeoffs of single static morphology designs, marking a substantial departure from existing paradigms.

To fully enable the loop closure grasping method, not only must a grasping mechanism design enable its open-loop form to fasten its tip to its base to transform into a closed loop, but its open-loop form must also enable the respective advantages for versatile grasp creation, and the closed-loop form must enable the respective advantages for simultaneously strong and gentle grasp holding. However, nearly all open-loop mechanisms with the tensile strength and bending compliance necessary to leverage the closed-loop topology do not have the articulation capabilities necessary to create versatile grasps because they require external work to bring its tip to its base (e.g., a caregiver must attach the ends of a sling to a base frame to close the loop around a patient’s body). In addition, open paths to create the desired stable grasping configuration around the object are often obstructed. For example, objects are almost always on a resting surface, which can prevent the mechanism from maneuvering underneath it to form a closed loop that can lift it from below. Other objects in the environment can also impede the necessary paths. These obstacles can substantially limit the variety of objects that can be grasped and configurations that can be achieved, especially in environments that are not specifically constructed to make the object accessible [e.g., not a factory or lab environment (*50*–*52*)]. To address these challenges for realizing loop closure grasping, we use soft growing inflated beam robots, or vine robots (*53*, *54*), as both an open-loop mechanism to navigate around the object through highly constrained environments to create versatile grasps and subsequently as a closed-loop mechanism with infinite bending compliance when deflated to hold simultaneously strong and gentle grasps.

The primary contribution of this work is the concept and design of loop closure grasping to enable simultaneously strong, gentle, and versatile grasps by leveraging the separate advantages of topologically open- and closed-loop morphologies. We present key principles that elucidate the fundamental benefits of sequencing topologically open-loop and closed-loop morphologies for safe, secure, and versatile grasping and an implementation of our architecture using inflated beam vine robots (*53*) to demonstrate this method of grasping. Our implementation consists of vine robots made from high-tensile strength materials with infinite bending compliance, pressurized bases that drive the vine robot growth, and tip-fastening mechanisms to close the loop. We demonstrate and characterize the ability to create versatile grasps and apply strong and gentle pulling and holding forces to grasp and lift a 6.8-kg kettlebell weight buried in a cluttered bin of parts, grasp via weaving an enclosure around a ball, grasp and lift a ring and a bucket with a handle via topologically interlocking Hopf links, grasp and pull containers from 3 m away, safely lift humans from a bed, lift and perform “in hand” manipulation with a cylinder, and grasp and lift a watermelon.

## RESULTS

### Loop closure grasping method for creating and holding grasps

To develop this method, we first decompose the grasping process into its functional stages and associated requirements and formalize a simple model of grasping mechanism morphology to define its topological classifications in the context of grasping. We decompose the process of grasping into the two stages of creating and subsequently holding the grasp. The functional requirements for these stages, respectively, are the following: (i) The grasping mechanism must articulate itself around the object to assume a desired, stable grasping configuration, and (ii) the grasping mechanism must continuously resist destabilizing forces during object manipulation to maintain the stable grasp without applying harmful contact forces. The key performance factor for the grasp creation stage that we consider is grasp versatility, which we define in terms of the variety and scale of objects it can grasp, the variety of stable grasping configurations it can deploy, and the variety of environments within which it can grasp these objects. The key performance factors for the second stage are how securely and gently it can hold heavy yet fragile objects. We define a heavy yet fragile object as one with a much higher weight relative to its allowable interaction forces. See text S1 for more detailed definitions.

**Fig. 2.**
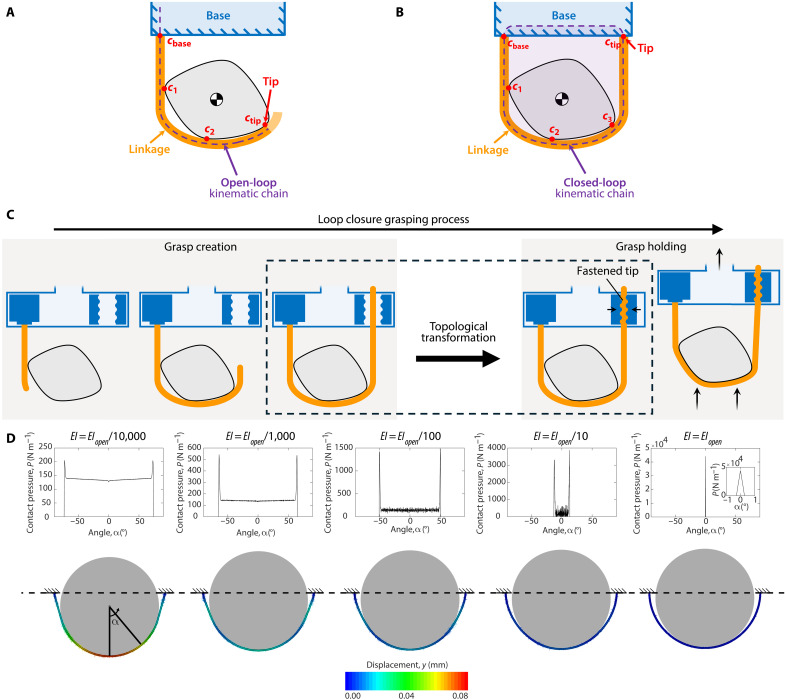
Topological model of grasping mechanisms and the loop closure grasping method. (**A** and **B**) Defining components and features of an open-loop and a closed-loop grasping mechanism, respectively. 𝑐 denotes a contact point. (**C**) Formal loop closure grasping method, sequenced in its three steps: open-loop grasp creation, topological transformation, and closed-loop grasp holding. (**D**) Pressure distributions between a circular object and five different closed-loop mechanisms with different flexural rigidities *EI*.

For the simple model of grasping mechanism morphology, the components of all grasping mechanisms can be categorized into three parts: the base, linkage, and tip (Fig. 2, A and B). The grasping mechanism is grounded to the base (typically fixed to some manipulator). The linkage articulates/activates to make contacts with the object to constrain the object’s position relative to the base. For our definition, we only care about the geometric path of the linkage, as it connects the base to the object. Thus, for generality, we abstract the linkage as a single kinematic serial chain (discrete or continuous) starting at the base and ending at the most distal contact point. We define this most distal contact point as the tip. We consider grasping devices with multiple serial kinematic chains as having multiple grasping mechanisms used together. For example, the grasping mechanism in human hands is the finger, of which five are grounded to the palm (the base). See text S2 for additional definitions regarding branching kinematic chains and mechanisms with geometries defined by two-dimensional (2D) manifolds.

We define the open- and closed-loop morphological classifications for grasping mechanisms based on their topology relative to the object. Our definitions involve the object’s location because we are interested in how the grasping mechanism interacts with the object, not just the mechanism itself. We consider a grasping mechanism to have a topologically open-loop morphology when its tip is not grounded (Fig. 2A). Thus, the tip is defined by the mechanism’s most distal contact point with the object or environment, and its position relative to the base is determined by the pose of the mechanism. We consider a grasping mechanism to have a topologically closed-loop morphology (Fig. 2B) if it satisfies the following two criteria: (i) The tip is grounded to the same system as the base, and (ii) the object is inside the loop created by the mechanism. See text S2 and fig. S1 for additional descriptions.

We now identify the key advantages and disadvantages of each topology for the different stages of grasping to inform how they can both be strategically used to circumvent the tradeoffs of previous single-morphology paradigms. In the “System design architecture and principles” section, we use these insights to formulate system design principles.

For the grasp creation stage, open-loop mechanisms are advantageous over closed-loop mechanisms for creating high grasp versatility because their tips are free to move around the object and environment into the desired grasping configuration. Given sufficient bending degrees of freedom (DOFs), they can theoretically navigate through any pathway wider than the mechanism’s cross section, as all points along its length can follow the same path [as established in continuum and hyperarticulated robotics literature (*42*, *53*, *55*–*58*)]. Conversely, the closed-loop topology limits grasp versatility because the tip is fixed. The entire mechanism must be maneuvered around the object at once, limiting the available paths that it can navigate through to create the desired grasp.

For the grasp holding stage, closed-loop mechanisms are advantageous over open-loop mechanisms for strong yet gentle grasping primarily because they can hold objects with theoretically infinite bending compliance (i.e., zero flexural rigidity) and passively distribute forces over a large contact area. Compliance enables gentle interactions with fragile objects as well established in soft robotics literature (*2*, *3*, *41*–*44*). All current open-loop grasping mechanism paradigms require some amount of flexural rigidity to stably hold objects (i.e., resist pulling/destabilizing forces), limiting the compliance of the grasp. Greater holding forces require greater rigidity, and, thus, a tradeoff exists between how strong and gentle the grasp can be. Closed-loop mechanisms do not require any flexural rigidity to hold objects; instead, the load can be borne entirely by their tensile strength. In text S3 and figs. S1 and S2, we prove that closed-loop mechanisms with zero flexural rigidity can stably hold objects, whereas open-loop grasping mechanisms with zero flexural rigidity, within current paradigms, fundamentally cannot. We also show that open-loop mechanisms with zero flexural rigidity cannot hold objects without applying additional torsional and friction shear forces (objects are generally stronger in compression than in shear), whereas closed-loop mechanisms can, and that this fundamental difference is a generalizable feature inherent to their topology across all possible morphologies. By eliminating flexural rigidity entirely, the closed-loop mechanism’s compliance when conforming to the object becomes theoretically infinite, eliminating bending reaction forces and, for convex objects, maximizing contact area. As shown in text S4 and fig. S3, we illustrate this benefit by simulating the contact pressure distributions of closed- and open-loop grasping mechanisms to compare their pressure distributions. Thus, the load capacity of closed-loop grasping mechanisms can be scaled up by increasing only its tensile strength without needing to increase flexural rigidity, eliminating the tradeoff between strong and gentle grasp holding inherent to open-loop mechanisms. Infinite bending compliance, in contrast to the high compliance used in previous grasping/harnessing mechanisms (*50*, *52*), is particularly advantageous because minimizing flexural rigidity in closed-loop suspensory mechanisms is always beneficial until it goes to zero. Grandgeorge *et al.* (*59*) showed that pressure concentrations increase at the two touch-down points on either side of the object as flexural rigidity increases. This is further shown in the simulation of contact pressure distributions of closed-loop mechanisms with increasing flexural rigidity in Fig. 2D (details in text S5 and fig. S4). Given that structures are generally much stronger/stiffer in tension than in bending, the mechanism’s thickness can also be kept low while maintaining high load capacities and thus can be made longer/wider for an allowable mass/volume to increase contact area and further decrease contact pressures. In addition, the closed-loop topology also enables notably higher grasp stability. For planar grasps, the mechanism topologically surrounds the entire object in a closed loop, and, thus, the object can only leave the grasp if the loop breaks. Similar to how multiple open-loop grasping mechanisms can be used in tandem to create a fully caged grasp in 3D space (*60*), multiple closed loops can also be combined to create this closure for 3D grasps (*50*).

Building on these elucidations of the grasping process, topological mechanism classifications, and their respective advantages and disadvantages, we now present the loop closure grasping method. Loop closure grasping is enabled by transforming the topology between the grasp creation and holding stages by fastening the mechanisms’ tips to their base frame. The method is illustrated in Fig. 2C.

The method is a sequence of three steps: (i) open-loop grasp creation, (ii) topological transformation, and (iii) closed-loop grasp holding. In the first step, the grasping mechanisms have an open-loop topology to perform the grasp creation stage. The mechanisms articulate around the object to assume the form for a desired stable closed-loop holding configuration. This includes positioning its tip, such that it can be fastened. The open-loop topology of the mechanism facilitates versatile grasp creation, enabling configurations for a wide variety of objects and environments. In the second step, the grasping mechanism’s topology is transformed to a closed loop by fastening its tip. To preserve the desired grasp configuration facilitated by the mechanism’s previously open-loop topology, the fastening method cannot alter the configuration. In the final step, the closed-loop grasping mechanisms perform the grasp holding stage. The mechanisms’ articulation becomes passive with minimal flexural rigidity to realize its “infinite” bending compliance. The closed-loop mechanisms’ configurations (created during the first step) must stably hold the object in a cradled suspended state (or a tightly cinched state by retracting its length) when the base is manipulated to pull/lift the object. The closed-loop topology facilitates strong and gentle holding of heavy yet fragile objects. To release the grasp, the tips of the mechanisms can be unfastened to transform the topology back to open-loop, at which point they can articulate (or retract) to remove themselves away from the object.

### System design architecture and principles

Here, we present a simple system design architecture and associated design principles for grasping systems that enable and realize the loop closure grasping method. This includes the system components, how they interrelate, and sets of principles for the components’ designs to satisfy the definitions, requirements, and goals of loop closure grasping. The formulation of this architecture and set of principles is informed by the grasping mechanism model, topological mechanism classifications, and their respective advantages and disadvantages presented in the previous section.

**Fig. 3.**
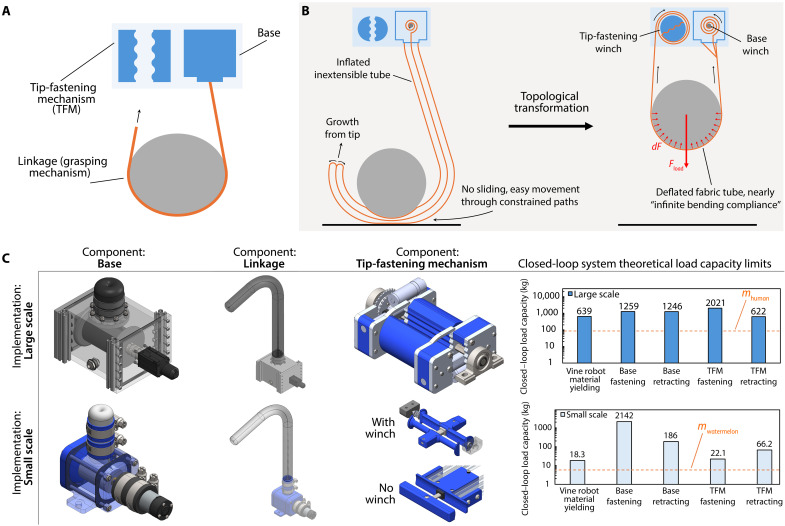
Loop closure grasping system design architecture and components design. (**A**) System design architecture with components labeled. TFM, tip-fastening mechanism. (**B**) Robotic implementation using soft growing inflated beams (i.e., vine robots), clamps, and winches. Our implementations of these mechanisms satisfy the design principles for each component and the overall architecture to enable and realize the loop closure grasping method. (**C**) Component designs for our large-scale and small-scale sets of system modules, and theoretical load capacity limits of the system due to vine robot material yielding (see scaling relationships in fig. S5) and the retraction force limits and fastening load capacities of the base and the tip.

The system design architecture is illustrated in Fig. 3A. On the basis of the previously presented simple grasping mechanism model, its components consist of (i) the base and (ii) the grasping mechanism (i.e., the linkage, mechanically grounded to the base), with the addition of (iii) a tip-fastening mechanism. Other components required to operate the linkage (e.g., actuators, transmissions, and controllers) can be stored in the base, as is often done in traditional gripper designs. The tip-fastening mechanism is fixed to the same frame as the base, such that they are both manipulated as a single unit. The workspace of the linkage’s tip must also contain the tip-fastening mechanism, such that they can engage to close the loop. Alternatively, tip-fastening mechanisms could be fixed to the tips of the grasping mechanisms, enabling two grasping mechanisms to fasten their tips together to create a single closed loop. Our results focus on the former case.

The design principles that we propose for each of these components are informed by the advantages and disadvantages of the open- and closed-loop morphologies for the grasp creation and holding stages presented in the previous section. They are as follows: The grasping mechanism design must satisfy the requirements and goals of both the grasp creation and holding stages, as it is the component that both actively creates the grasp configuration and holds the object. Thus, it must be able to function as an open-loop and closed-loop grasping mechanism and transform between them by fastening its tip to the same frame as its base.

To create the grasp, the grasping mechanism must articulate itself into the desired grasping configuration without any additional external work when in its open-loop form. To enable versatile grasps, its open-loop form should have high bending DOFs (ideally continuum) and a large configuration/work space to navigate through complex paths to achieve a high variety of configurations. It should also have a slender cross section to enable navigation through constrained environments (e.g., through environmental obstacles) and objects [e.g., through a closed loop in the object to create a Hopf link (*61*)]. While not fundamental to the architecture, additional beneficial functionalities include the ability to extend (so that the mechanism can follow the path of the tip, minimizing the space it moves through), the ability to pass between gaps smaller than its own width to navigate through especially constrained paths (e.g., get between objects and their resting surface), and the ability to make safe soft contact with the object while articulating around it (to enable configurations that start in contact with the object).

To enable strong yet gentle grasp holding in its closed-loop form, the grasping mechanism should have a high passive tensile strength to bear heavy forces and negligible flexural rigidity (i.e., infinite bending compliance) for gentle distribution of contact pressures. It should have high passive bending DOF (ideally continuum) and a large passive configuration space in its closed-loop form as well to enable high shape conformability and maximize contact area with the object (especially when convex). An additional beneficial, but not fundamental, functionality includes the ability to be retracted at its base and/or tip to enable active tightening of the loop to apply pulling and cinching forces.

The base and the tip-fastening mechanism do not actively create the grasping configuration or directly hold the object, and so their purposes are primarily to enable the grasping mechanism’s functionalities for loop closure grasping. To securely ground the grasping mechanism, the base design must be able to bear the same or greater loads than its closed-loop tensile strength. The tip-fastening mechanism design must be able to reversibly fasten the tip of the grasping mechanism to engage and release the closed-loop hold, as well as bear the same or greater loads than the grasping mechanism. The fastening mechanism design cannot impose any design requirements onto the grasping mechanism that would impede its performance and/or satisfaction of its respective design principles (e.g., requiring the grasping mechanism to have some fastening feature at its tip that would limit its ability to navigate through obstacles). An additional beneficial, but not fundamental, functionality for both the base and the tip-fastening mechanism is active, high-strength, and reversible retraction of the grasping mechanism’s length to tighten the closed loop and/or apply pulling forces.

### Grasping system implementation with soft growing inflated beams

To realize and demonstrate the loop closure grasping concept, we created a set of modular components to be used to create loop closure grasping systems (Fig. 3). We developed two sets of component modules, one for large-scale grasping and one for small-scale grasping, with modules for the base, linkage, or tip-fastening mechanism. Their different designs and constructions are described in detail in Materials and Methods and text S6. A single set of these modules can be used to create a single-loop grasping system with one grasping mechanism. Multiple systems can be mounted to the same ground frame and used in tandem to create more sophisticated grasping systems. Our design implementation for each component is as follows (Fig. 3): a high tensile strength pneumatically driven soft growing robot [i.e., a vine robot (*53*)] as the linkage, a high-strength motorized base, and a high-force/torque tip-fastening winch device.

We use vine robots as the grasping mechanism because their distinct structure and mechanics fully satisfy, to an exceptional degree, all of our associated system design principles. Vine robots, which are long tendril-like soft inflated robotic structures that navigate their environments via pressure-driven “growth” from their tip, have an open-loop topology. They can also navigate through cluttered spaces without sliding friction [including between objects and their resting surface (*62*)] due to their tip-everting growth mechanism, grow to long lengths to wrap around large and/or far away objects, and grow into 3D configurations against gravity due to their light weight (*63*). In addition, when deflated, vine robots can have a high tensile strength and nearly infinite bending compliance and conformability, given proper material selection, precisely the characteristics needed to leverage the closed-loop topology for strong and gentle grasp holding. While vine robots have previously only been used as open-loop mechanisms (*54*, *64*), our systems incorporate tip-fastening mechanisms that secure the tips of vine robots to transform their topology to closed-loop, enabling their tensile strength and infinite bending compliance to be leveraged for grasp holding. Thus, by navigating around the object, anchoring its tip onto its base via a tip-fastening mechanism, and deflating, vine robots can be used to create loop closure grasps.

Similar to most bases for vine robots (*54*, *64*), our base design consists of a pressurized box that drives the vine robot growth and an internal motorized winch to wind/unwind its inner material to control growth and apply pulling forces. This method of pulling via winding up the material is possible because of its infinite bending compliance and enables work multiplication (*65*) to lift heavy loads over long distances using relatively low-power actuators. The load-bearing inner material is permanently fastened to the winch, the strength of which is made sufficiently high (while maintaining a compact diameter) by ensuring that the inner material is always wrapped over some minimum angle so the fastening force is always magnified via capstan friction.

Our tip-fastening mechanism design consists of a clamp-winch device, as shown in Fig. 3C. For some of our small-scale demonstrations that did not require pulling from the tip, we designed and used clamps without the winch function for compactness. To reversibly fasten and pull on the vine robot, the tip-fastening winch closes its clamp after the tip has been inserted and then rotates to wind up its length. Unlike many past vine robot–related works (*66*–*68*), this clamping method enables the vine robot to be fastened directly, without requiring any tip-mounted devices that would impede its ability to navigate through highly constrained paths into complex self-supported configurations. For its high reversible fastening strength, capstan friction from wrapping around the winch cannot be used here because the wrapping angle is zero when the vine robot is first fastened. However, capstan friction is still used through a wave pattern implemented in the clamp surface design that wraps the vine robot around a series of circular curved segments, as shown in fig. S6. The holding force for a segment of the vine robot along one curve is determined by the load capacity of the segment along the previous curve. Thus, the loading force is magnified at an exponential rate over the total wrapping angle across the series of curves, just like in the standard Euler-Eytelwein formula (*69*). In text S7 and fig. S6, we show that the load capacity of the wave-patterned clamp can be conservatively estimated as follows

$$T_{\text{load}}=\mu F_{\text{clamp}}e^{\mu \left(n+1\right)\theta_{c}}$$ (1)

where μ is the coefficient of friction between the vine robot inner material and itself (assuming that the load is only on the inner material, which is the case when the vine robot length is also retracted by the base), $F_{\text{clamp}}$ is the clamping force, *n* is the effective number of curve segments on one of the clamp surfaces, and $\theta_{c}$ is their central angle.

The pulling strength of both the base and the tip-fastening winch depends on the gear ratio of their transmission and the maximum radius of their winch, including the thickness of the wound material. The retraction mechanism in both devices is simply the winch spooling up the length of the vine robot.

The strength of the overall grasp is determined by the weakest of all of these factors. The theoretical limits that each component imposes on the system, assuming that it is the bottleneck, are shown in Fig. 3C (details in text S6). Currently, the bottleneck factors for the large-scale and small-scale systems’ load capacities are the tip-fastening winch retraction force limit and vine robot tensile strength, yielding a 622- and 18.3-kg capacity for a single closed-loop vine robot mechanism, respectively. The large-scale load capacity bottleneck could be increased while keeping the motor torque capacity the same by reducing the diameter of the base winch or increasing the transmission’s gear reduction. The small-scale bottleneck could be increased by changing the membrane material or thickness.

### Grasp versatility

**Fig. 4.**
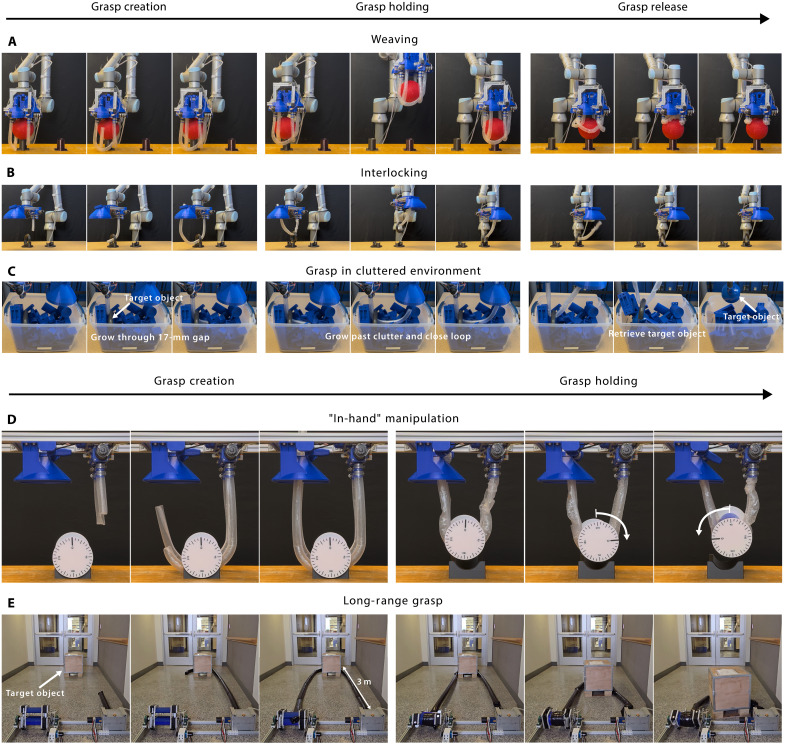
Grasp versatility demonstrations. (**A**) Weaving around an object to secure it. (**B**) Creating Hopf link by interlocking with ring. (**C**) In hand manipulation of a grasped object. (**D**) Grasping object in a cluttered environment. (**E**) Grasping an object resting 3 m away from grasping mechanism.

To demonstrate the grasp versatility enabled using an initially open-loop morphology, we performed three different grasps: (i) grasping a ball with four vine robots in a woven configuration, (ii) grasping a ring and a bucket handle via interlocking closed loops with a single vine robot, and (iii) grasping a kettlebell weight in a cluttered environment (as well as a vase resting on a table). To demonstrate the ability to create grasps with different configurations, we used four vine robots to create a woven configuration around a ball to grasp and lift it. As previously demonstrated in (*50*), the entanglement of the woven warp and weft threads create a mechanically stable 3D enclosure to grasp the object, which they achieved by twisting flexible closed loops such that they closed down onto the object from an initially sprawled configuration. In our demonstration, the vine robots were grown directly into the closed configuration, as shown in Fig. 4A and movie S1, allowing for grasps in constrained environments. Using this configuration, we grasped and lifted a 178-mm-diameter rubber ball, which would otherwise be more difficult to stably lift with an unwoven configuration. The grasp was successful and withstood inertial disturbances from manipulation. To illustrate the ability to release the grasp, we subsequently released the tips of the vine robots and retracted their lengths back into their bases, placing the ball in a new location.

To demonstrate the ability to grasp objects with different topologies, we used both our small-scale and large-scale grasping system prototypes to create interlocked Hopf links (*70*) between a single vine robot and objects with permanently closed loops, namely, a ring and the handle of the bucket. As shown in Fig. 4B and movie S2, using an initially open-loop mechanism to navigate through the object before closing the loop, we create grasps that would be topologically impossible with a permanently open- or closed-loop grasping mechanism. Assuming that neither loop breaks, this grasp is theoretically infinitely stable to disturbances. To the best of our knowledge, this is the first demonstration of robotic grasping of any kind with this specific topological configuration.

To demonstrate the ability to grasp objects in difficult environments, we grasped a kettlebell in a cluttered environment (e.g., a bin filled with loose parts) and grasped a glass vase resting on a table. Creating versatile grasps with permanently closed-loop grasping mechanisms can be challenging when obstacles exist in the environment because the entire closed loop must maneuver around the object at once [e.g., (*50*, *51*), lassos]. Conversely, open-loop mechanisms can theoretically navigate through any path wider than its cross section (*53*). Further, vine robots can even pass through paths narrower than its own width. As shown in Fig. 4D and movie S3, we used a single vine robot to successfully grasp and lift the kettlebell surrounded by various objects of different shapes and sizes. The smallest gap that the vine robot had to navigate through was ~17 mm wide, roughly 35% smaller than the 26-mm diameter of the vine robot. In addition, to illustrate the ability to create loop closure grasps for objects resting on a surface with no gaps (i.e., the resting surface fully blocks the desired path for closing the loop around the object), a glass vase grasped and lifted from a table, as shown in Fig. 1D and movie S4. Three vine robots grow their tips through the interface between the vase and table from opposite directions to balance the horizontal pushing force that each exerts on the object. The vine robots navigate past the obstruction of the resting surface without a preexisting gap to close the loop and gently hold the vase for lifting.

In addition to enabling loop closure grasping, vine robots have benefits for achieving typically difficult grasps due to (i) the inextensibility of its membrane material and (ii) its high length extension ratio. The first characteristic is beneficial for bearing tensile loads substantially greater than the payloads of human-safe robots (*71*) (as we later demonstrate in the “Strong and gentle grasping and lifting” section), expanding the versatility of object weights that can be lifted. Its inextensibility also enables the transmission of forces over long distances, as initially hypothesized in (*53*).

The high extension ratios of vine robots enable the grasping mechanism to grasp objects that are both (i) substantially far away from its base and (ii) substantially larger than its base. This is because, to create a stable grasp, the mechanism must be able to sufficiently reach around the object to apply the constraining contacts/forces at the appropriate locations, which may not be feasible for objects substantially larger than the grasping mechanism or far away from the base frame.

To demonstrate the ability of the vine robot grasping mechanism to grasp and pull objects at distances much further away than its own length, we performed loop closure grasps on boxes 3.0 m away and pulled it to the base, as shown in Fig. 4E and movie S5. The distance over which the container was grasped and pulled was 896% longer than the length of the vine robot base system (0.335 m), with the vine robot growing to a total length of over 6.7 m. The combined cross-sectional area of the bounding boxes of the motorized base and tip-fastening winch is 0.180 m^2^, whereas the cross-sectional area of the larger box in the horizontal plane is 135% greater at 0.244 m^2^. Having grown over 6.7 m long, the vine robot theoretically could have also grasped a larger nearby object with a circular cross-sectional area over 1985% greater than that of the base and winch.

**Fig. 5.**
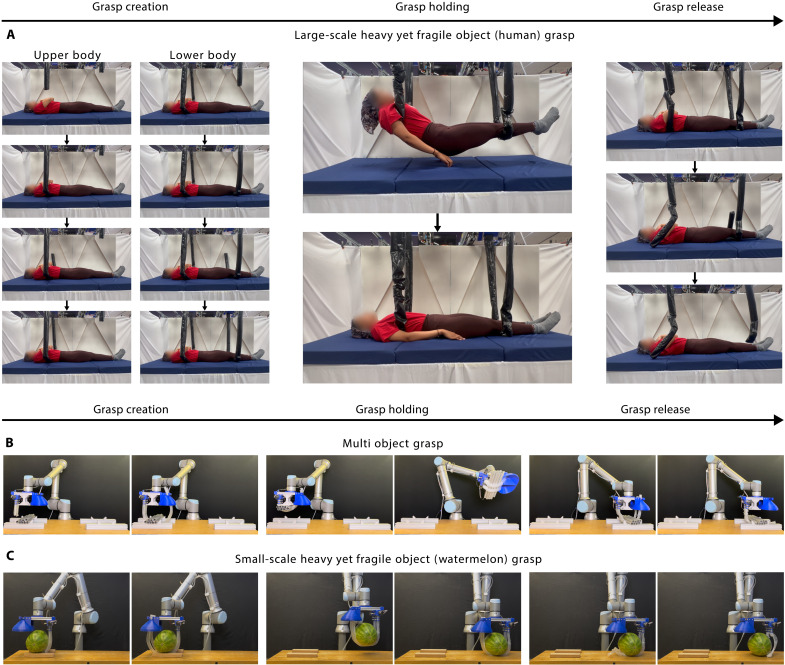
Harnessing and lifting demonstrations. (**A**) Harnessing and lifting a human participant with large-scale grasping implementation system. (**B**) Simultaneously grasping and transferring multiple objects. (**C**) Grasping and transferring a heavy yet fragile object (watermelon).

We also demonstrate multiobject grasps (as shown in Fig. 5B and movie S6) by wrapping around and cinching a pile of pipes by retracting the vine robots’ length back into the base, while the tip is still fastened. The closed-loop morphology encloses the pipes into a bound space, and tightening the vine robots creates a force closure grasp that secures the pipes together via friction, removing the grasp’s dependence on gravity to keep the object in a cradled state. The system then manipulates the grasped objects to a target location, where the vine robots invert to smoothly release the grasp without sliding friction. This approach further highlights the system’s adaptability to handle irregularly shaped or clustered objects while maintaining secure grip and reliable release.

Leveraging the continuous flexibility of the deflated vine robots and the fact that the winches can feed excess length between them, our systems can also manipulate objects in hand. Assuming sufficient static friction to prevent slipping, the vine robot material can be fed from the base to the tip, or vice versa, while keeping its total length constant, such that the object rotates without translating (analogous to a belt driving an idler pulley). To demonstrate this, we grasped and lifted a cylinder, deflated the vine robot, and extended/retracted it by the tip and base winches to rotate it while holding in mid-air (as shown in Fig. 4C and movie S7).

### Strong and gentle grasping and lifting

We demonstrate the ability to safely grasp and lift heavy yet fragile objects through loop closure grasping by handling a human with our large-scale system (as shown in Fig. 5A and movie S8). Specifically, two vine robot base/tip device subsystems were fixed above a human (weight, 74.1 kg; height, 170.2 cm) lying on a bed and used to grasp and lift their full weight to illustrate the system’s ability to (i) create a stable enveloping grasp around a body without a preexisting open path for that configuration and (ii) safely and stably lift a heavy yet fragile object.

The system was able to close the loop around the participant in a stable configuration (wraps around and supports the body from below) by growing its tip into the interface between the body and the bed, frictionlessly tunneling its way to the other side to place itself under the body, and growing back upward via obstacle-aided navigation (*72*) into the tip-fastening winch (see text S8 and fig. S7 for calculations of the path uncertainty for navigation into the tip-fastening winch). The system successfully lifted the full weight of the participant ~25 cm above the bed without causing any excess harm or discomfort (see text S9 for participant responses). We also demonstrate releasing the grasp by unfastening the vine robot tip and retracting it back from under the body.

To further evaluate the “safety” of using vine robots to create loop closure grasps around humans on a resting surface, we measured the contact pressures experienced by a 79.4-kg life-sized manikin as a single vine robot grew underneath it. The manikin was instrumented with a soft capacitive sensor sleeve, and the normal contact pressure distribution was measured over the duration of the vine robot growing under the body (see Materials and Methods for details).

**Fig. 6.**
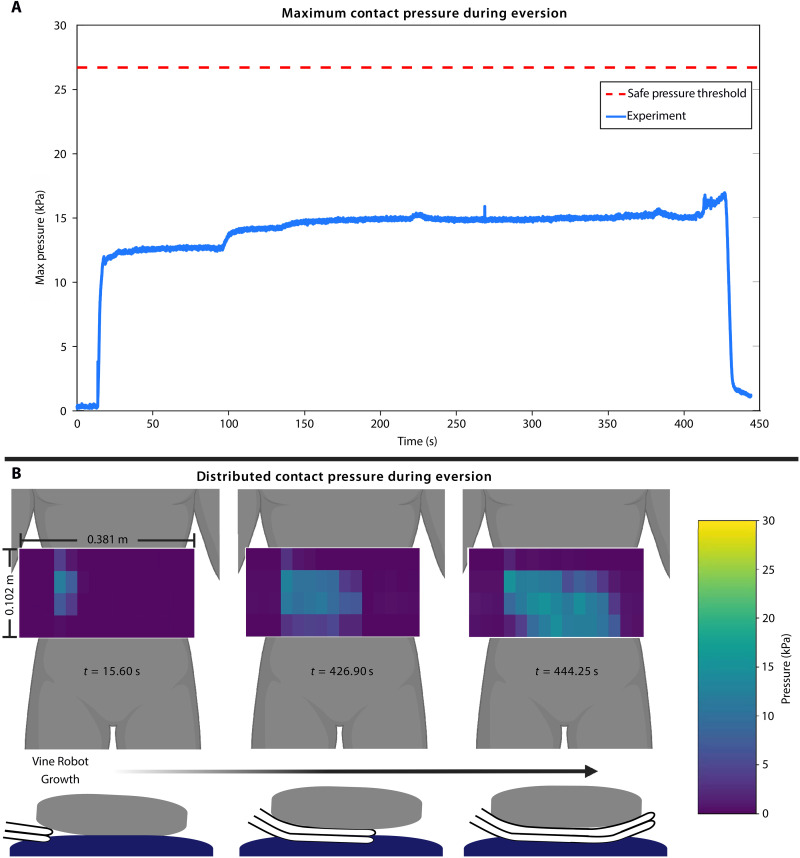
Pressure distributions from life-sized manikin weight (79.4 kg) lying on bed, while vine robot grows under back. (**A**) Maximum contact pressure during vine robot growth between the body and the bed. The peak pressure measured over the entire duration was 16.95 kPa. (**B**) During vine robot growth between the body and the bed at time *t* = 15.60, 110.40, and 319.34 s.

As shown in Fig. 6 and movie S9, as expected, the maximum pressure concentrations occur at the contact between the body and the inflated vine robot. The peak pressure measured over the entire duration was 16.95 kPa. This is substantially lower than the peak pressures experienced by patients during standard transfers in medical patient slings (>26.7 kPa) (*73*), showing that the interaction with the vine robot during the grasping process is safe for the human body.

We further demonstrate the ability to safely grasp heavy yet fragile objects with our small-scale system by grasping and lifting a watermelon (as shown in Fig. 5C and movie S10) and a 1.0-kg glass vase (as previously described in the “Grasp versatility” section and shown in Fig. 1D and movie S4). For the former, the system was positioned above a 5.9-kg watermelon (29 cm by 20 cm by 20 cm), and two vine robots independently grew under and around the watermelon into the tip-fastening mechanisms to close the loops. The entire device was then raised to successfully lift the watermelon without damage.

## DISCUSSION

Loop closure grasping transitions between both open- and closed-loop topologies to leverage each of their respective benefits for the different stages of the grasping process. This marks a substantial departure from traditional design paradigms that only make use of a single topology, in that it relieves the need for the open-loop form to satisfy the functional requirements of maintaining safe and effective grasps and the need for the closed-loop form to satisfy the functional requirements for creating versatile grasps.

Decoupling each topology’s involvement from their lesser-suited stage of the grasping process enables their full potential to be used to best satisfy the stage they are best suited for. This is particularly highlighted by our proof that open-loop grasping mechanisms fundamentally cannot hold objects with infinite bending compliance (without applying notable additional torsion and friction forces and not at all within current grasping paradigms), while closed-loop mechanisms can. Grasping mechanisms designed with permanently open-loop morphologies to enable versatile grasping will always be limited in the gentle compliance it can achieve relative to closed-loop morphologies. Conversely, open-loop mechanisms can theoretically navigate around objects through any path that their tip can pass through (as demonstrated in continuum and hyperarticulated robotics, assuming that its tip is its widest point, and growing robotics, as shown in this work and past literature), whereas permanently closed-loop mechanisms are fundamentally hindered by needing to navigate their entire linkage around the object at once due to lacking a free tip.

By using vine robots with tip-fastening mechanisms, we enable the full benefits of both topologies and the ability to transition between them to use them in the respective stages of grasping that they are beneficial for. Vine robots are particularly beneficial for creating versatile grasps due to their exceptional ability to navigate through highly constrained environments via eversion-based growth, including through spaces that are smaller than its tip [as shown in literature (*53*, *54*, *64*) and our cluttered environment and human lifting demonstrations] and their highly articulable continuum structure. Their high extension ratio coupled with their compact storability also enables scalable grasping of larger objects, with substantial increases in available vine robot length only requiring small increases in the size of the base. Their light weight and soft structure also allow for safe navigation around fragile objects. Because of the flexible and inextensible construction of vine robot membranes, their morphology changes from a tube to a sheet when deflated. Similar to slings, belts, straps, and hammocks used in industry, this morphology lends well for achieving high tensile strength and virtually zero flexural rigidity, enabling it to fully leverage the closed-loop topology when its tip is fastened for safely holding high loads with “infinite compliance.”

Our method and system design principles can theoretically be realized using any type of robotic open-loop mechanism that can fasten its tip to its base (*33*, *74*–*76*). Different mechanisms can present different advantages and disadvantages, and, thus, this work presents loop closure grasping as a general method that can be implemented with any type of mechanism that satisfies our proposed architecture and principles. While vine robots are particularly beneficial for realizing loop closure grasping, they also present some challenges including easily disturbed growth trajectory away from the desired path toward the tip-fastening mechanism and difficult to instrument with sensors for closed-loop position control. Investigations of loop closure grasping system designs using different mechanisms can further elucidate best practices for implementation in different applications with different goals and requirements.

We demonstrate how our loop closure grasping paradigm enables versatile grasping of objects that are heavy yet fragile, topologically challenging, large in scale, and in constrained environments, all of which have traditionally been challenging for robots. In addition to advancing performance, these benefits are also conceptually meaningful for soft robotic grasping, in that the infinite compliance maximizes one of the core benefits of soft robotics, and the high pull-out force overcomes one of its most substantial traditional limitations (compared to rigid grasping mechanisms). Further, we envision that this paradigm will broaden the practical applicability of robotic grasping to a wider range of potential high-impact applications. Robotics could be made more applicable to many industries that require handling of humans, such as eldercare, care for people with disabilities, patient care in hospitals, emergency medical response, and search and rescue. While our current demonstration of lifting a human with only two vine robots may not provide the most comfortable experience, future work can investigate the use of more, wider, and/or branching vine robots to distribute the load over larger contact areas. Other applications involving heavy yet fragile objects include handling livestock, agricultural harvesting, heavy manufacturing, large cargo handling, construction, infrastructure repair, and ship/aircraft wreckage recovery. Grasping versatility is also important for these applications, as the robot must adapt to the various requirements for each presented task. In aerospace applications, long-extending loop closure grasping mechanisms could be used to collect in-orbit space debris, transfer payloads between spacecraft, or retether detached astronauts. In wearable applications, loop closure mechanisms could be used to automatically fasten and secure clothing and devices to the body. In fast-paced warehouse sorting, the passive strength, stability, and compliance of loop closure grasps could enable more dynamic manipulation by mitigating the effects of potentially destabilizing or damaging high inertial accelerations.

To transition from the open- to closed-loop topologies, the tip of the open-loop mechanism must be able to navigate its tip to the base to be fastened. While we successfully demonstrate this using preforming methods to program the path through which the vine robots grow, a limitation of our study is that we did not use actuated steering or bending mechanisms to actively articulate its tip into the fastening mechanism. Adding active articulation [which has been studied extensively for robots with open-loop morphologies (*54*)] could markedly improve the robustness and adaptability of loop closure grasping systems to the levels of dexterity enabled by current continuum and hyperarticulated robots. Robust implementations of active articulation for vine robots in three dimensions at larger scales remain an open research question that stands to greatly improve their use in loop closure grasping. In addition, the safety of interaction between our systems and the objects (e.g., a human) while bearing their weight could be further investigated. In-depth theoretical analyses of the pressure distributions and contact mechanics of loop closure grasping systems could be further elucidated and enable the optimization of future grasping mechanisms using this paradigm.

## MATERIALS AND METHODS

### Construction of loop closure grasping system components

The vine robots used in our large-scale systems are constructed by heat-sealing the thermoplastic polyurethane–coated sides of 70-denier nylon ripstop fabric (Seattle Fabrics Inc.) in a tubular shape to provide a high tensile strength, airtight, inexpensive, and lightweight construction with negligible flexural rigidity. For the small-scale systems, we construct the vine robots using low-density polyethylene (ULINE), which has a lower but still sufficient tensile strength of 10 MPa (*77*). We use preforming to navigate the vine growth direction, as well as obstacle-aided passive steering for the human grasping demonstration [both are standard methods established in vine robot literature (*54*)].

The base consists of a motorized winch inside of a pressurized chamber with an air-tight collar to fasten the base of the vine robot (Fig. 3). For the large-scale systems, the chamber is made from aluminum to provide a strong grounding frame on which the winch can exert high pulling forces. The collar and winch are 3D printed out of carbon fiber–infused nylon with fiberglass continuous fiber reinforcements. The winch has a steel shaft core and is driven by a brushless dc motor (Rev Robotics LLC) with a 100:1 ratio gearbox. For the small-scale systems, the chamber and collar are 3D printed out of polylactic acid (PLA) as one piece. The winch has a steel shaft core and is driven by a brushed dc motor with a 455:1 ratio gearbox (Robotzone LLC).

Our implementation of the tip-fastening mechanism for the large-scale system is a winch with an embedded clamp with smoothly corrugated jaws to fasten and wind up load-bearing vine robots steel hex shaft and two linear actuators (Eco-Worthy), with partially threaded bolts used to reinforce the 3D printed parts. For the small-scale system, both clamps and tip-fastening winches are used for different demonstrations (Fig. 3C). They are both constructed primarily from 3D-printed PLA and a single linear actuator (UYGALAXY). Please refer to text S6 for additional material specifications and construction details for all components.

### Human grasping and lifting demonstration participant

A healthy female adult (age, 25 years; weight, 74.1 kg; height, 170 cm) participated in the experiment (Fig. 5A). The participant was informed about the experiment and provided informed consent before participation. The vine robots were pressurized to 24.1 kPa and turned 90° before navigating under the participant and were pressurized to 13.8 kPa and turned 45° after emerging from the opposite side. They grew into the tip-fastening winches via obstacle-aided navigation (*72*) using surface features in the adjacent wall. The vine robots were then fully depressurized, wound up by both ends to lift and hold the participant 25 cm above the mattress, and unwound to gently lower her back down. Before the lift, the participant was instructed to keep their body slack except for raising their head to avoid injury as they were lowered. The participant’s upper body is lifted first to ensure an upright position. Then, their lower body is lifted. The participant rated her comfort throughout the experiment using Likert scales. All experimental procedures were approved by the respective Institutional Review Boards of the Massachusetts Institute of Technology and Stanford University.

### Contact pressure during growth under human experiment

For the experiment illustrated in Fig. 6, a 79.4-kg life-sized manikin (Simulaids International Association of Fire Fighters Rescue Randy) was used. Its weight is distributed to match the average US adult weight distribution. To measure contact pressures, it was outfitted with a custom soft capacitive sensor array created by Pressure Profile Systems (Los Angeles, CA, USA). Please refer to text S10 for further details.

## Supplementary Materials

### The PDF file includes:

Texts S1 to S10 Figs. S1 to S7 Legends for movies S1 to S10 Legends for data S1 to S3 References

## References

[1] Z. Samadikhoshkho, K. Zareinia, F. Janabi-Sharifi, “A brief review on robotic grippers classifications,” in *2019 IEEE Canadian Conference of Electrical and Computer Engineering (CCECE)* (IEEE, 2019), pp. 1–4.

[2] J. Shintake, V. Cacucciolo, D. Floreano, H. Shea, Soft robotic grippers. Adv. Mater. 30, 1707035 (2018). 10.1002/adma.201707035 29736928

[3] J. Hughes, U. Culha, F. Giardina, F. Guenther, A. Rosendo, F. Iida, Soft manipulators and grippers: A review. Front. Robot. AI 3, 69 (2016).

[4] K. Tai, A.-R. El-Sayed, M. Shahriari, M. Biglarbegian, S. Mahmud, State of the art robotic grippers and applications. Robotics 5, 11 (2016).

[5] C. Piazza, G. Grioli, M. G. Catalano, A. Bicchi, A century of robotic hands. Annu. Rev. Control Robot. Auton. Syst. 2, 1–32 (2019).

[6] A. Billard, D. Kragic, Trends and challenges in robot manipulation. Science 364, eaat8414 (2019). 31221831 10.1126/science.aat8414

[7] S. C. deWit, P. A. Williams, *Fundamental Concepts and Skills for Nursing* (Elsevier, 2013).

[8] Z. Feng, Global convergence: Aging and long-term care policy challenges in the developing world. J. Aging Soc. Policy 31, 291–297 (2019). 31154942 10.1080/08959420.2019.1626205

[9] S. A. Lavender, K. M. Conrad, P. A. Reichelt, A. K. Kohok, J. Gacki-Smith, Designing ergonomic interventions for emergency medical services workers—Part III: Bed to stairchair transfers. Appl. Ergon. 38, 581–589 (2007). 17070768 10.1016/j.apergo.2006.08.002

[10] A. E. Fürst, R. Keller, M. Kummer, C. Manera, B. Von Salis, J. Auer, R. Bettschart-Wolfensberger, Evaluation of a new full-body animal rescue and transportation sling in horses: 181 horses (1998–2006). J. Vet. Emerg. Crit. Care 18, 619–625 (2008).

[11] M. Samdal, H. Eiding, L. Markengbakken, J. Røislien, M. Rehn, M. Sandberg, Time course of hoist operations by the search and rescue helicopter service in Southeast Norway. Wilderness Environ. Med. 30, 351–361 (2019). 31653552 10.1016/j.wem.2019.06.004

[12] D. Penrose, *Occupational Therapy for Orthopaedic Conditions* (Springer, 1993).

[13] K. E. Norman, A. Pepin, M. Ladouceur, H. Barbeau, A treadmill apparatus and harness support for evaluation and rehabilitation of gait. Arch. Phys. Med. Rehabil. 76, 772–778 (1995). 7632134 10.1016/s0003-9993(95)80533-8

[14] H. Srivatsan, A. V. Myagerimath, V. G. Duffy, “A systematic review of collaborative robots in ergonomics,” in *Digital Human Modeling and Applications in Health, Safety, Ergonomics and Risk Management* (Springer, 2024), pp. 282–297.

[15] R. Bostelman, J. Albus, N. Dagalakis, A. Jacoff, “RoboCrane project: An advanced concept for large scale manufacturing,” in *Proceedings of the AUVSI Conference*, Orlando, FL, 1 July 1996 (AUVSI, 1996).

[16] R. Hoffman, H. H. Asada, Precision assembly of heavy objects suspended with multiple cables from a crane. IEEE Robot. Autom. Lett. 5, 6876–6883 (2020).

[17] J. O. Glerum, S. Kelly, *Stage Rigging Handbook* (Southern Illinois Univ. Press, ed. 3, 2007).

[18] B. Kelechava, “ASME B30.9-2021: Slings,” *ANSI Blog*, 20 January 2022; https://blog.ansi.org/ansi/asme-b30-9-2021-slings/.

[19] D. Ibekwe, “It took 2 cranes to lift the 41-tonne plane that skidded off an icy runway in Turkey,” *Business Insider*, 20 January 2018; www.businessinsider.com/2-cranes-recover-plane-skidded-off-runway-turkey-trabzon-airport-2018-1.

[20] Crane Lifting Slings, “Bridles and assemblies,” *Tri-State Rigging Equipment*; https://tsriggingequipment.com/crane-lifting-slings-bridles-assemblies.

[21] B. Roy, A. Basmajian, H. Asada, “Maneuvering a bed sheet for repositioning a bedridden patient,” in *2003 IEEE International Conference on Robotics and Automation (ICRA)* (IEEE, 2003), pp. 2224–2229.

[22] M. Mooney, “BMW scales up use of 3D-printed robot grippers for car assembly,” *Robotics and Automation*, 24 May 2024; www.roboticsandautomationmagazine.co.uk/news/assembly/bmw-scales-up-use-of-3d-printed-robot-grippers-for-car-assembly.html.

[23] E. E. Phillips, “Massive robots keep docks shipshape,” *Wall Street* Journal, 27 March 2016; www.wsj.com/articles/massive-robots-keep-docks-shipshape-1459104327.

[24] T. Mukai, S. Hirano, H. Nakashima, Y. Kato, Y. Sakaida, S. Guo, S. Hosoe, “Development of a nursing-care assistant robot RIBA that can lift a human in its arms,” in *2010 IEEE/RSJ International Conference on Intelligent Robots and Systems (IROS)* (IEEE, 2010), pp. 5996–6001.

[25] C. Loh, H. Tsukagoshi, “Pneumatic Big-hand gripper with slip-in tip aimed for the transfer support of the human body,” in *2014 IEEE International Conference on Robotics and Automation (ICRA)* (IEEE, 2014), pp. 475–481.

[26] R. Baines, S. K. Patiballa, J. Booth, L. Ramirez, T. Sipple, A. Garcia, F. Fish, R. Kramer-Bottiglio, Multi-environment robotic transitions through adaptive morphogenesis. Nature 610, 283–289 (2022). 36224418 10.1038/s41586-022-05188-w

[27] E. Sihite, A. Kalantari, R. Nemovi, A. Ramezani, M. Gharib, Multi-modal mobility morphobot (M4) with appendage repurposing for locomotion plasticity enhancement. Nat. Commun. 14, 3323 (2023). 37369710 10.1038/s41467-023-39018-y PMC10300070

[28] J. Sun, E. Lerner, B. Tighe, C. Middlemist, J. Zhao, Embedded shape morphing for morphologically adaptive robots. Nat. Commun. 14, 6023 (2023). 37758737 10.1038/s41467-023-41708-6 PMC10533550

[29] T. F. Nygaard, C. P. Martin, J. Torresen, K. Glette, D. Howard, Real-world embodied AI through a morphologically adaptive quadruped robot. Nat. Mach. Intell. 3, 410–419 (2021).

[30] T. J. Cairnes, C. J. Ford, E. Psomopoulou, N. Lepora, An overview of robotic grippers. IEEE Potentials 42, 17–23 (2023).

[31] J. Hernandez, M. S. H. Sunny, J. Sanjuan, I. Rulik, M. I. I. Zarif, S. I. Ahamed, H. U. Ahmed, M. H. Rahman, Current designs of robotic arm grippers: A comprehensive systematic review. Robotics 12, 5 (2023).

[32] D. Prattichizzo, J. C. Trinkle, Grasping, in *Springer Handbook of Robotics* (Springer, 2016), pp. 955–988.

[33] K. Becker, C. Teeple, N. Charles, Y. Jung, D. Baum, J. C. Weaver, L. Mahadevan, R. Wood, Active entanglement enables stochastic, topological grasping. Proc. Natl. Acad. Sci. U.S.A. 119, e2209819119 (2022). 36215466 10.1073/pnas.2209819119 PMC9586297

[34] E. Brown, N. Rodenberg, J. Amend, A. Mozeika, E. Steltz, M. R. Zakin, H. Lipson, H. M. Jaeger, Universal robotic gripper based on the jamming of granular material. Proc. Natl. Acad. Sci. U.S.A. 107, 18809–18814 (2010).

[35] F. Aljaber, A. Hassan, T. Abrar, I. Vitanov, K. Althoefer, “Soft inflatable fingers: An overview of design, prototyping and sensorisation for various applications,” in *2023 IEEE International Conference on Soft Robotics (RoboSoft)* (IEEE, 2023), pp. 1–6.

[36] J. Shintake, S. Rosset, B. Schubert, D. Floreano, H. Shea, Versatile soft grippers with intrinsic electroadhesion based on multifunctional polymer actuators. Adv. Mater. 28, 231–238 (2016). 26551665 10.1002/adma.201504264

[37] J. Qu, Z. Yu, W. Tang, Y. Xu, B. Mao, K. Zhou, Advanced technologies and applications of robotic soft grippers. Adv. Mater. Technol. 9, 2301004 (2024).

[38] G. He, C. Sparks, N. Gravish, Grasping and rolling in-plane manipulation using deployable tape spring appendages. Sci. Adv. 11, eadt5905 (2025). 40203100 10.1126/sciadv.adt5905 PMC11980837

[39] S. Li, D. M. Vogt, D. Rus, R. J. Wood, Fluid-driven origami-inspired artificial muscles. Proc. Natl. Acad. Sci. U.S.A. 114, 13132–13137 (2017). 29180416 10.1073/pnas.1713450114 PMC5740677

[40] V. Cacucciolo, J. Shintake, H. Shea, “Delicate yet strong: Characterizing the electro-adhesion lifting force with a soft gripper,” in *2019 2nd IEEE International Conference on Soft Robotics (RoboSoft)* (IEEE, 2019), pp. 108–113.

[41] C. Majidi, Soft robotics: A perspective—Current trends and prospects for the future. Soft Robot. 1, 5–11 (2014).

[42] C. Laschi, B. Mazzolai, M. Cianchetti, Soft robotics: Technologies and systems pushing the boundaries of robot abilities. Sci. Robot. 1, eaah3690 (2016). 33157856 10.1126/scirobotics.aah3690

[43] M. Cianchetti, C. Laschi, A. Menciassi, P. Dario, Biomedical applications of soft robotics. Nat. Rev. Mater. 3, 143–153 (2018).

[44] O. Yasa, Y. Toshimitsu, M. Y. Michelis, L. S. Jones, M. Filippi, T. Buchner, R. K. Katzschmann, An overview of soft robotics. Annu. Rev. Control Robot. Auton. Syst. 6, 1–29 (2023).

[45] A. Fox, “New research suggests humans invented string at least 120,000 years,” *Smithsonian Magazine*, 10 July 2020; www.smithsonianmag.com/smart-news/study-suggests-humans-invented-string-least-120000-years-ago-180975286/.

[46] The Editors of Encyclopaedia Britannica, Ed., “sling,” *Encyclopedia Britannica*, 29 May 2013; www.britannica.com/technology/sling.

[47] M. C. Langley, T. Suddendorf, Mobile containers in human cognitive evolution studies: Understudied and underrepresented. Evol. Anthropol. 29, 299–309 (2020). 32744760 10.1002/evan.21857

[48] T. Suddendorf, K. Kirkland, A. Bulley, J. Redshaw, M. C. Langley, It’s in the bag: Mobile containers in human evolution and child development. Evol. Hum. Sci. 2, e48 (2020). 37588341 10.1017/ehs.2020.47 PMC10427442

[49] “Hoisting and rigging fundamentals for riggers and operators,” (TR244C, Rev. 5, US Department of Energy, 2002); www.energy.gov/sites/prod/files/2014/01/f6/HoistingRigging_Fundamentals.pdf.

[50] G. Kang, Y.-J. Kim, S.-J. Lee, S. K. Kim, D.-Y. Lee, K. Song, Grasping through dynamic weaving with entangled closed loops. Nat. Commun. 14, 4633 (2023). 37532695 10.1038/s41467-023-40358-y PMC10397280

[51] L. Manes, S. Fichera, H. Fakhruldeen, A. I. Cooper, P. Paoletti, A soft cable loop based gripper for robotic automation of chemistry. Sci. Rep. 14, 8899 (2024). 38632348 10.1038/s41598-024-59372-1 PMC11024125

[52] K. Barhydt, H. H. Asada, A high-strength, Highly-flexible robotic strap for harnessing, lifting, and transferring humans. IEEE Robot. Autom. Lett. 8, 2110–2117 (2023).

[53] E. W. Hawkes, L. H. Blumenschein, J. D. Greer, A. M. Okamura, A soft robot that navigates its environment through growth. Sci. Robot. 2, eaan3028 (2017). 33157883 10.1126/scirobotics.aan3028

[54] L. H. Blumenschein, M. M. Coad, D. A. Haggerty, A. M. Okamura, E. W. Hawkes, Design, modeling, control, and application of everting vine robots. Front. Robot. AI 7, 548266 (2020). 33501315 10.3389/frobt.2020.548266 PMC7805729

[55] M. Russo, S. M. H. Sadati, X. Dong, A. Mohammad, I. D. Walker, C. Bergeles, K. Xu, D. A. Axinte, Continuum robots: An overview. Adv. Intell. Syst. 5, 2200367 (2023).

[56] X. Dong, D. Axinte, D. Palmer, S. Cobos, M. Raffles, A. Rabani, J. Kell, Development of a slender continuum robotic system for on-wing inspection/repair of gas turbine engines. Robot. Comput. Integr. Manuf. 44, 218–229 (2017).

[57] J. Burgner-Kahrs, D. C. Rucker, H. Choset, Continuum robots for medical applications: A survey. IEEE Trans. Robot. 31, 1261–1280 (2015).

[58] E. Del Dottore, A. Mondini, N. Rowe, B. Mazzolai, A growing soft robot with climbing plant–inspired adaptive behaviors for navigation in unstructured environments. Sci. Robot. 9, eadi5908 (2024). 38232147 10.1126/scirobotics.adi5908

[59] P. Grandgeorge, T. G. Sano, P. M. Reis, An elastic rod in frictional contact with a rigid cylinder. J. Mech. Phys. Solids 164, 104885 (2022).

[60] S. Makita, Y. Maeda, “3D multifingered caging: Basic formulation and planning,” in *2008 IEEE/RSJ International Conference on Intelligent Robots and Systems (IROS)* (IEEE, 2008), pp. 2697–2702.

[61] D. Rolfsen, *Knots and Links* (American Mathematical Society, 2003).

[62] T. Nakamura, H. Tsukagoshi, “Soft pneumatic manipulator capable of sliding under the human body and its application to preventing bedsores,” in *2018 IEEE/ASME International Conference on Advanced Intelligent Mechatronics (AIM)* (IEEE, 2018), pp. 956–961.

[63] L. H. Blumenschein, N. S. Usevitch, B. H. Do, E. W. Hawkes, A. M. Okamura, “Helical actuation on a soft inflated robot body,” in *2018 IEEE International Conference on Soft Robotics (RoboSoft)* (IEEE, 2018), pp. 245–252.

[64] M. M. Coad, L. H. Blumenschein, S. Cutler, J. A. R. Zepeda, N. D. Naclerio, H. El-Hussieny, U. Mehmood, J.-H. Ryu, E. W. Hawkes, A. M. Okamura, Vine robots. IEEE Robot. Autom. Mag. 27, 120–132 (2020).

[65] E. W. Hawkes, C. Xiao, R.-A. Peloquin, C. Keeley, M. R. Begley, M. T. Pope, G. Niemeyer, Engineered jumpers overcome biological limits via work multiplication. Nature 604, 657–661 (2022). 35478234 10.1038/s41586-022-04606-3

[66] S.-G. Jeong, M. M. Coad, L. H. Blumenschein, M. Luo, U. Mehmood, J. H. Kim, A. M. Okamura, J.-H. Ryu, “A tip mount for transporting sensors and tools using soft growing robots,” in *2020 IEEE/RSJ International Conference on Intelligent Robots and Systems (IROS)* (IEEE, 2020), pp. 8781–8788.

[67] A. M. Kübler, S. U. Rivera, F. B. Raphael, J. Förster, R. Siegwart, A. M. Okamura, “A multi-segment, soft growing robot with selective steering,” in *2023 IEEE International Conference on Soft Robotics (RoboSoft)* (IEEE, 2023), pp. 1–7.

[68] R. Jitosho, S. Simón-Trench, A. M. Okamura, B. H. Do, “Passive shape locking for multibend growing inflated beam robots,” in *2023 IEEE International Conference on Soft Robotics (RoboSoft)* (IEEE, 2023), pp. 1–6.

[69] L. Euler, Remarques sur l’effet du frottement dans l’équilibre. Mémoires de l’académie des sciences de Berlin 18, 265–278 (1769).

[70] C. C. Adams, *The Knot Book: An Elementary Introduction to the Mathematical Theory of Knots* (American Mathematical Society, 2004).

[71] F. Sherwani, M. M. Asad, B. Ibrahim, “Collaborative robots and industrial revolution 4.0 (IR 4.0),” in *2020 International Conference on Emerging Trends in Smart Technologies* (*ICETST*) (IEEE, 2020), pp. 1–5.

[72] J. D. Greer, L. H. Blumenschein, R. Alterovitz, E. W. Hawkes, A. M. Okamura, Robust navigation of a soft growing robot by exploiting contact with the environment. Int. J. Robot. Res. 39, 1724–1738 (2020).

[73] M. J. Peterson, J. A. Kahn, M. V. Kerrigan, J. M. Gutmann, J. J. Harrow, Pressure ulcer risk of patient handling sling use. J. Rehabil. Res. Dev. 52, 291–300 (2015). 26237005 10.1682/JRRD.2014.06.0140

[74] M. Jiang, Q. Yu, N. Gravish, “Vacuum induced tube pinching enables reconfigurable flexure joints with controllable bend axis and stiffness,” in *2021 IEEE 4th International Conference on Soft Robotics (RoboSoft)* (IEEE, 2021), pp. 315–320.

[75] M. Wang, X. Dong, W. Ba, A. Mohammad, D. Axinte, A. Norton, Design, modelling and validation of a novel extra slender continuum robot for in-situ inspection and repair in aeroengine. Robot. Comput. Integr. Manuf. 67, 102054 (2021).

[76] M. Kaneko, N. Kanayama, T. Tsuji, Active antenna for contact sensing. IEEE Trans. Rob. Autom. 14, 278–291 (1998).

[77] O. Szlachetka, J. Witkowska-Dobrev, A. Baryła, M. Dohojda, Low-density polyethylene (LDPE) building films – Tensile properties and surface morphology. J. Build. Eng. 44, 103386 (2021).

[78] A. Rodriguez, M. T. Mason, S. Ferry, From caging to grasping. Int. J. Robot. Res. 31, 886–900 (2012).

[79] H. Hanafusa, H. Asada, Stable prehension of objects by the robot hand with elastic fingers. Trans. Soc. Instrum. Control Eng. 13, 370–377 (1977).

[80] O. G. Osele, K. Barhydt, N. Cerone, A. M. Okamura, H. Harry Asada, “Tip-clutching winch for high tensile force application with soft growing robots,” in *2024 IEEE International Conference on Robotics and Automation (ICRA)* (IEEE, 2024), pp. 9362–9368.

[81] N. Agharese, “Modeling and interfacing with vine robots,” thesis, Stanford University, Stanford, CA (2023).

