# Supplementary material for: Loop closure grasping: Topological transformations enable strong, gentle, and versatile grasps
Source: Sci Adv. 2025 Dec 10;11(50):eady9581. doi: 10.1126/sciadv.ady9581 (PMC12694038; doi:10.1126/sciadv.ady9581)
Supplement: Supplementary file 1 — Texts S1 to S10 Figs. S1 to S7 Legends for movies S1 to S10 Legends for data S1 to S3 References [file sciadv.ady9581_sm.pdf]

Science Advances
AAAS

Supplementary Materials for

Loop closure grasping: Topological transformations enable strong, gentle, and versatile grasps

Kentaro Barhydt *et al.*

Corresponding author: Kentaro Barhydt, kbarhydt@mit.edu

Sci. Adv. **11**, eady9581 (2025)
DOI: 10.1126/sciadv.ady9581

The PDF file includes:

Texts S1 to S10
Figs. S1 to S7
Legends for movies S1 to S10
Legends for data S1 to S3
References

Other Supplementary Material for this manuscript includes the following:

Movies S1 to S10
Data S1 to S3

S1. Additional context for problem definitions section

Here we present a formalization of the task of harnessing (i.e. grasping in loop closure, grasping in weak form closure or in a grasping cage *(78)*) and pulling heavy yet fragile objects.

The key factors for success are the security and gentleness of the grasp.

i. **Grasping**: We consider the object to be grasped if it is mechanically stable inside of the grasp such that some level of work is required to remove it from the stable region, and the security is quantified as the amount of work required to destabilize it *(79)*. The grasp must be performed independently, in that no external physical effort from either the object or an outside agent is required.
ii. **Pulling**: We consider the object to be pulled if the subsequent manipulation forces displace it toward the base of the manipulator over some distance.
iii. **Gentleness**: We quantify the "gentleness" of the interaction as the ratio between the maximum pressures exerted onto the object and the maximum allowable contact pressures the object can experience without harm/damage.
iv. **Heavy Yet Fragile**: We define a heavy yet fragile object as one with a substantially high weight relative to its allowable interaction forces, and quantify this characteristic as the ratio of its weight to its maximum allowable contact pressures.

To address the versatility of applications in which a mechanism can achieve this task, we also define grasping versatility in terms of three factors:

i. The variety and scale of objects it can grasp,
ii. The variety of grasping configurations it can deploy (i.e. configuration space of the grasping mechanism), and
iii. The variety of environments within which it can grasp these objects.

The second and third factors are included to account for the fact that a mechanism's ability to successfully grasp an object is also dependent on the accessibility of the object in the given environment and the desired grasping configurations for the given task.

S2. Additional context for closed-loop vs. open-loop morphologies

We present a general framework for representing grasping mechanisms in the "Closed-Loop vs. Open-Loop Morphologies" section. Additionally, for grasping mechanisms with branching kinematic chains, we consider each chain, starting at one of the tips and ending at either the base or a point along another chain, to be its own grasping mechanism. Thus, for open-loop mechanisms, the number of mechanisms equals the number of tips. If a grasping mechanism is grounded to another grasping mechanism, we consider that proximal mechanism to serve as the distal mechanism's base. If the full geometric paths that the grasping mechanism creates relative to the object cannot be represented with a single one-dimensional serial chain (e.g. textiles), then it can instead be abstracted as a two-dimensional manifold (a surface), with its base and tip taking the same definitions as the grounded points and distal points of contact, respectively. Again, because we are interested only in the paths that the mechanism creates relative to the object to bridge the base and its points of contact, paths created by mechanisms with kinematics

that could be represented with a three-dimensional geometry (e.g. particle jamming gripper *(34)*) can be simplified as just a one- or two- dimensional manifold for our definition.

We also define the closed-loop morphology as meeting the following two criteria: (1) the tip is grounded to the same system as the base, and (2) the object is inside of the loop created by the mechanism. For the first criterion, if the tip (the most distal contact point along the mechanism) is defined by its fixture to the base instead of contact with the object, then the path through the kinematic chain of the grasping mechanism (including the base) is now closed. If the tip is grounded to a different frame than the base, it still satisfies criteria one as long as the positions of both grounds are fixed relative to each other or manipulated relative to each other by the same system (e.g. both fixed to the world frame, both fixed to the same manipulator, different manipulators on same robot, different robots in the same multi-robot control system). The ground segment that closes the kinematic chain from the tip to the base is defined by the shortest path between them. If the tip is grounded to a proximal point along itself (e.g., a lasso), then the length of the kinematic chain distal to that contact point is considered the closed-loop grasping mechanism. Following the definitions of bases posed for branching mechanisms in the previous paragraph, the length of the kinematic chain serves as the base to this closed-loop mechanism, because it grounds both its proximal end and its tip. We include the second criterion in our definition because the grasping morphology is concerned with how the mechanism interacts with the object, not just with the mechanism itself. Many grasping mechanisms include closed-loop kinematic chains within them (e.g. parallel linkage gripper), but the object is not inside of those closed loops, and thus their tips are not grounded and are still considered to have an open-loop grasping morphology. Closed-loop mechanisms surround the object in at least one plane, which creates a fully caged grasp in the two-dimensional space of that plane. Similar to how multiple open-loop grasping mechanisms can be used in tandem to create a fully caged grasp in three-dimensional space *(78)*, multiple closed-loop mechanisms can be used to accomplish this as well.

S3. Closed-loop grasping morphologies enable grasps with infinite bending compliance (proof)

Here we present a logical proof that current open-loop grasping mechanism paradigms cannot stably pull on objects if they have zero flexural rigidity, whereas closed-loop mechanisms with zero flexural rigidity can. We also show that, across all possible mechanism designs, open-loop mechanisms with zero flexural rigidity fundamentally cannot stably pull on objects without applying substantial additional torsional and friction shear forces, whereas closed-loop mechanisms with zero flexural rigidity can. To illustrate how we arrive at these specific claims, this section is presented in three steps: (1) we present the proof that this fundamental different exists between open-loop and closed-loop mechanisms, (2) we describe a single counter example of open-loop mechanisms with zero flexural rigidity that can stably hold objects, and (3) we describe the caveats of this counterexample and its separation from current design paradigms to arrive back to our initially stated claims.

Open-loop mechanisms constrain the object through geometric and/or force constraints *(4, 32)*. We consider the two respective types of grasping within existing paradigms: enveloping (caging, form closure) and pinching (force closure), as shown in Fig. S1A and Fig. S1E.

In the enveloping case, the grasping mechanism pulls on the object by applying a force F behind it. To reach behind it, the mechanism must extend past the side of the object. Thus, a moment arm d will always exist between the base of the mechanism and the point of force application, creating an internal bending moment M. This applies for all mechanisms, open- and closed-loop. For open-loop morphologies, if the mechanism has no flexural rigidity, it cannot resist the moment M, and thus will bend away from the object until it is no longer behind it. Some level of flexural rigidity, whether structural or induced through actuation, is required to resist M. Similar logic can be used to evaluate the pinching case (Fig. S1E-F), in which open-loop mechanisms generate friction forces F_f in the pulling direction by applying normal forces N. The object must be pulled over some distance, so a moment arm d will always exist. Without any flexural rigidity, the open-loop mechanism cannot resist the resulting moments M and will bend away from the object until it is no longer in contact. Therefore, in both the enveloping (form closure, caging) and pinching (force closure) cases, open-loop mechanisms with zero flexural rigidity cannot stably hold and pull grasped objects.

For comparison in both cases, we evaluate closed-loop mechanisms as two pseudo-open loops on either side of the object with their tips fastened together (Fig. 2C-D, Fig. S1G-H). The segmentation point between them can be chosen arbitrarily. If these pseudo-open loops apply pushing forces F or frictional forces F_f to pull the object, bending moments M will still be generated. However, if a pseudo-open loop mechanism cannot independently resist M and begins to bend away from the object, the other mechanism is pulled further around the object. Therefore, even if the mechanisms have no flexural rigidity and deform due to contact forces, they will always surround the object in a stable caged grasp.

Given this proof, here we provide an alternative derivation of the principle through an evaluation of a differential segment of a grasping mechanism. As the closed-loop mechanism is pulled, it fully conforms to the object and becomes taught. Here, we show that the reaction forces from the base pulling both ends of the loop in tension can balance the internal moments, enabling stable pulling. Fig. S2 shows a free body diagram of a differential segment of a grasping mechanism at one of its contact points with the grasped object. dF denotes the differential normal force from the object contact, c denotes the effective contact point at which dF is applied, R denotes the radius of curvature, p_1 and p_2 denote the end points at the left and right of the differential segment, respectively, $d\phi$ subtends arc $p_1\,p_2$, T_1 and T_2 denote the tensile forces applied onto the differential segment from the adjacent segments at points p_1 and p_2, respectively, and M_{R1} and M_{R2} denote the bending reaction moments in the differential segment due to its flexural rigidity EI, respectively. The subscripts 1 and 2 denote the side closer to the base and tip, respectively. M_{R1} and M_{R2} are generated in reaction to the change in local curvature, i.e. bending with respect to the adjacent segments. As the force of the object onto the mechanism pushes it to bend back, the curvature of the mechanism decreases, causing the reaction moment M_R to increase according to the bending moment equation: $M_R = (\kappa - \kappa_0)E\,I$.

The balances of the force and moment about point p_1 are:

$$\Sigma F_x = (T_2 - T_1)\cos\frac{d\phi}{2} \tag{S1}$$

$$\Sigma F_y = (T_1 + T_2)\sin\frac{d\phi}{2} - dF \tag{S2}$$

$$\Sigma M_{p1} = M_{R1} - M_{R2} + \left(T_2 \sin\frac{d\phi}{2}\right)\left(2R \sin\frac{d\phi}{2}\right) - dF\left(R \sin\frac{d\phi}{2}\right) \quad \text{(S3)}$$

For the grasp to be stable, the forces and moments of all differential segments of the grasping mechanism must be balanced when at steady-state. We can therefore ignore inertial effects.

If the mechanism has zero flexural rigidity ($E\ I = 0$), then $M_{R1} = M_{R2} = 0$. Therefore, the moment balance becomes:

$$\Sigma M_{p1} = \left(T_2 \sin\frac{d\phi}{2}\right)\left(2R \sin\frac{d\phi}{2}\right) - dF\left(R \sin\frac{d\phi}{2}\right) \quad \text{(S4)}$$

For open-loop grasping mechanisms within current paradigms, $T_2 = 0$ and because the tip of the mechanism is free, so there is no tension pulling the segment toward the tip. This yields:

$$\Sigma M_{p1} = -dF\left(R \sin\frac{d\phi}{2}\right) \quad \text{(S5)}$$

The moment is not balanced (unstable) as long as some contact force is applied. Therefore, open-loop mechanisms with zero flexural rigidity cannot stably pull on objects.

However, in closed-loop mechanisms, the tip is grounded, and thus can supply a reaction tensile force T_2 to the entire mechanism. Thus, for a differential segment, the force and moment balances take the form of equations S1, S2, and S4. If the mechanism has zero flexural rigidity and $M_{R1} = M_{R2} = 0$, the total moment can still be balanced ($\sum M_{p1} = 0$).

As stated earlier, while the fundamental difference between open-loop and closed-loop mechanisms that we present here exists within current paradigms of grasping, within the span of all possible mechanisms, there is a hypothetical counterexample for a potential system of open-loop mechanisms with zero flexural rigidity that could theoretically hold an object. This system consists of multiple continuum open-loop mechanisms with zero flexural rigidity (e.g., a rope or whip) hanging down with one side grounded to the base frame. If one of the "ropes" is wrapped around the object (e.g., a cylinder, for simplicity) multiple times and we assume that there is some weight at its tip positioned such that it stably holds itself down onto the cylinder, then the friction holding force experienced by the other end of the "rope" can be amplified substantially via the capstan effect. If the cylinder could not rotate, then it can be held in a suspended state by the "rope" due to the capstan friction, which would also apply a torsional moment onto the cylinder. However, the cylinder can rotate, and thus this moment would cause it to rotate counter to the wrapping direction and fall as the "rope" unravels. To prevent this, additional "ropes" could also be wrapped around the cylinder with some wrapped in the opposite direction, such that the applied torsional moments cancel out on the object. The capstan friction still holds up the weight of the object and the "rope" wrappings do not unravel, making the hold stable.

The existence of this counterexample shows that, while our proof holds for existing grasping mechanism paradigms, it does not hold for the entire span of all possible mechanism systems. However, this counterexample necessitates that additional shear forces are applied onto the object. The capstan friction must be high enough to hold the object, applying frictional shear to the object surface. The opposing moments from the "ropes" wrapped in opposite directions also apply a torsion onto the object proportional to its weight. These are applied in addition to normal compressive forces, which are necessary for the capstan friction to be applied. Conversely,

closed-loop grasping mechanisms with zero flexural rigidity can hold objects while only applying compression forces, without torsion or friction. Thus, it is still true that, across all possible mechanism designs, open-loop mechanisms with zero flexural rigidity cannot stably pull on objects without applying substantial additional torsional and friction shear forces, whereas closed-loop mechanisms with zero flexural rigidity can. Given that materials are generally substantially stronger in compression than in shear, the utilization of the closed-loop topology still provides a fundamental advantage for gently holding heavy yet fragile objects over this counterexample of using multiple frictional open-loop “ropes”.

S4. Closed-loop vs. open-loop mechanism pressure distribution finite element analysis

Fig. S3 shows the results of a closed-loop vs. open-loop mechanism pressure distribution finite element analysis. Fig. S3 shows the results of a closed-loop vs. open-loop mechanism pressure distribution finite element analysis. The finite element models used for this work were built in Abaqus CAE 2021. The models are 2D Deformable bodies with a Solid, Homogeneous section. The closed-loop with negligible flexural rigidity was modeled using an Elastic material model with directional rigidity, where the stiffness along the loop (E1) was set to 125 MPa, and the flexural rigidity (E2) was set to 0.1 MPa. The closed-loop mechanism with flexural rigidity and open-loop mechanism with flexural rigidity both used isotropic, non-directional material models with 125 MPa stiffness. The stiffness of the sphere was set to that of steel for all three cases (E = 200 GPa). The mass density for the loops was set to $5 \times 10{-}9$ tonne/mm^3 for the loops and $7.9 \times 10{-}9$ tonne/mm^3 for the spheres. We used 2D tetrahedral meshes for both bodies, with a global size of 20 mm.

The simulation was run in two separate steps, both Static General. In the first step, we used thermal expansion to ensure that the simulation would converge while the sphere was in full contact of the loops. For all three simulations, we used an expansion coefficient of $5 \times 10{-}4$ on the steel spheres and applied a Predefined Temperature Field with a magnitude of 8.2 which we ramped throughout the step. We used General Contact on both bodies without friction and using “Hard” Contact for normal contact. In the second step, we applied gravity on both bodies using a Smooth Step. We applied Encastre boundary conditions on the fixed ends of the loops.

In post processing, we extracted CPRESS, the magnitude of the net contact normal force, from the last frame of each simulation.

S5. Finite element analysis of effects of varying flexural rigidity of a Closed-Loop mechanism on its pressure distribution on idealized object

Fig. S4 shows the results of a finite element analysis of the effects of varying flexural rigidity of a Closed-Loop mechanism on its pressure distribution on idealized object. Fig. S4 shows the results of a finite element analysis of the effects of varying flexural rigidity of a Closed-Loop mechanism on its pressure distribution on idealized object. Closed-loop mechanisms holding circular objects were simulated in using finite element methods to observe the relationship between the contact pressure distribution and the flexural rigidity of the mechanism. All

simulations were developed and performed in Abaqus. The mechanisms were modeled as a two-dimensional deformable planar shell, with a neutral radius of 0.51 m, an initial radius of 0.5 m, a constant second moment of inertia, and a Poisson's ratio of 0.2. The Young's modulus was parametrically swept over the range of mechanisms simulated, from 10 kPa to 10 GPa. Their densities were set to scale linearly with Young's modulus (based on typical scaling behavior from experiments, i.e., as shown on a standard Ashby plot) with values ranging from 101 kg/m^3 to 106 kg/m^3. The object was modeled as a 2D rigid body discrete wire object with a radius of 0.45 m. The contacts were defined as interactions between the top surface of the mechanism and the perimeter of the object. All contact interactions are set to be frictionless. Contact interactions were tracked – including pressures, forces, and areas (length in 2D) – node-wise across the top/inner surface of the mechanism as a field output for post-processing. A fixed-fixed semicircle beam boundary condition was applied. The downward force was also applied uniformly to the boundary of the object, ramped from zero to the final weight. For the solver, static steps were implemented throughout the closed loop simulation procedure.

S6. Loop closure grasping mechanism implementation details

The loop closure grasping system concept we describe is comprised of three main components: (1) the base, (2) the grasping mechanism(kinematic chain that transitions from open-loop to closed- loop topology), and (3) the tip-fastening mechanism grounded to the same body and/or system as the base (Fig. 1C). The grasping mechanism navigates its tip around the object into the tip-fastening mechanism to create a loop closure grasp. Although this framework can be realized through many different implementations, we describe the one used for the system shown in Fig. 3, which uses vine robots as the grasping mechanism body, as well as winches in both the base and tip-fastening mechanism to retract the length of the closed loop to apply lifting forces.

The security of the closed-loop holding of objects is dependent on the configurations of the deflated vine robots relative to the object. Notable factors include the number of vine robots used, the locations of the bases, and the locations of the contact points with the object. Once these closed-loop holding configurations were determined, the configurations of the open-loop inflated vine robots and the locations of their bases and tip-fastening mechanisms were designed such that deflating, fastening, and lifting the vine robots would result in the desired closed-loop holding configurations. To ensure that the target closed-loop holding configuration was secure, they were designed based on the following conditions.

For grasps formed by interlocking two closed loops (Hopf link, e.g., ring lifting demo and cluttered environment demo), only one vine robot was required to securely grasp the object, without considerations for the configuration other than creating the Hoph link. This is because the two closed-loops of the vine robot and the object will remain topologically interlocked regardless of their shapes as long as neither loop is broken.

For the remaining grasps in which the object is cradled under gravity by the vine robots (without any topological interlocking), the closed-loop holding configurations were designed such that two primary conditions were met to ensure balance. First, the vertical projection of the object center of mass must lie within the support polygon of the vine robot contact points such that the

net moment on the object is zero. The support polygon is the smallest convex polygon in the horizontal plane that encloses the contact points that constrain the pose of the object. For the cradling grasps demonstrated in this paper, the kinematic constraints applied onto the object's pose by a single closed-loop vine robot can be defined by just the touch-down and lift-off points. Both points are a fixed distance from their respective base or tip-fastening mechanism as long as the object does not roll relative to the vine robots (which holds true when the grasp is in steady-state). The remaining contact points are free to rotate (with the object) about the axis intersecting the touch-down and lift-off points, and thus do not constrain any additional degrees of freedom. A support polygon requires at least three vertices (more than a single line) for the center of mass to lie within it, so at least two vine robots were used for all of the cradling grasps.

Second, the tension vectors that the vine robots apply onto the object must lie within their corresponding friction cones to avoid slipping. For the woven grasp demonstration, with an object geometry that makes aligning the tensioned deflated vine robots with the feasible friction cones especially difficult, the vine robots were woven together such that they held each other together and prevented slipping off of the object. The specific weaving pattern used in our demonstration was based on the pattern shown in *(50)*.

For the human grasping and lifting demonstration, the articulated and pliable nature of the human body necessitated an additional consideration for designing the closed-loop holding configurations: the vine robots must contact the body at joints that act as local height minima for the point of contact along the body, so that the vine robot cannot slide further up the surface of the body (i.e., the body cannot slide down relative to the vine robot). If the weight of the body segments on both sides of a joint supported by a vine robot are large enough, then the adjacent segments of the body fall below it. Thus, the joint becomes the locally highest point, and the geometry of the body in contact with the vine robot at that point is concave. The gravitational potential energy of the body is at a local minimum because the vine robots cannot slide away from the joints without raising their height. Additionally, the center of mass of the body is lower than the vine robot contact points. Thus, the closed-loop holding configuration is secure. In our human lifting demonstration, the two joints supported by the vine robots were the shoulder joints and knee joints. For the shoulder joints, the torso and arms locally fell below the shoulder, and thus the shoulder acted as a concave local height minimum. For the knee joints, the thighs and shins fell below the knees, and thus the knees acted as a concave local height minimum. This leveraging of concave points is similarly utilized in caging grasps *(78)*.

For the demonstrations using the large scale loop closure grasping system, we selected a commercially available thermoplastic polyurethane (TPU) -coated 70-denier nylon ripstop fabric (Seattle Fabrics Inc., WA, USA) for its high tensile strength (13.1 N/mm), light weight (170 g/m^2), negligible flexural rigidity, and the ability to heat-seal its TPU-coated side to itself to create strong air-tight seals. The fabric was formed into the tubular shape of the vine robots by sealing two sheets together using a Vetron 5064 ultrasonic welder (Vetron Typical Europe GmbH, Kaiserslautern, Germany) for convenience, although they could also have been made using impulse sealers *(64)*. When inflated, the vine robots are 76.2 mm in diameter, and 119.7 mm wide when deflated and flattened. For the demonstrations using the small scale loop closure grasping system, we construct the vine robots using 0.15mm thick Low Density Polyethylene (ULINE, North America) that is 64.7 mm in diameter when inflated and 101.6 mm wide when

deflated and flattened. The growth direction of vine robots can be actuated, generally, by four different methods: Distributed Strain, Concentrated Strain, Tip-Localized Strain and Preforming *(54)*. This work utilizes preforming for actuating the growth direction of the vine. The scaling relationships between the tensile load capacity of the open-loop vine robots with respect to their diameter for different membrane materials are illustrated in Fig. S6. The open-loop load capacity of a vine robot is calculated as $T_{OL} = \sigma_{y,mem}\pi D$, where $\sigma_{y,mem}$ is the tensile yield strength per unit width of the membrane material (N/mm) and D is the diameter of the vine robot (mm). The theoretical load capacity of the vine robot in the closed-loop configuration is twice that of the open-loop configuration.

The base consists of a motorized winch inside of a pressurized chamber with an air-tight collar to fasten the base of the vine robot to, as illustrated in Fig. 3C. For the human lifting and long range demo, the pressurized chamber is made from aluminum to provide a strong grounding frame on which the winch can exert high forces onto the vine robot. The winch consists of a 31.8 mm diameter cylinder 3D printed from carbon-fiber infused nylon filament, with a 12.7 mm wide steel hex shaft core to withstand the high forces and torques applied onto the vine robot. The end of the vine robot inner material is fastened to the cylinder using cloth-backed adhesive tape (Gorilla Glue, Inc., Ohio, USA), and the winch was controlled such that the material is wrapped around by at least 2.5 rotations at all times to maintain a high capstan friction load capacity. For the fastening strength of the base, the point of connection is between the inner material of the vine robot and the motorized winch that winds/unwinds it. When lifting an object, the winch winds the inner material up while the outer material crumples its length, and thus the inner material bears the entire load. We leverage capstan friction to implement high-strength fastening by ensuring that the vine robot inner material was always wrapped around the winch over some minimum angle value so the holding force is always magnified. This amplification can be conservatively estimated using the Euler-Eytelwein formula: $T_{load} = T_{hold}e^{\mu\theta}$, where T_{load} is the load capacity, T_{hold} is the holding force at the tip of the vine robot inner material, μ is the coefficient of friction, and θ is the wrapping angle. The holding force implemented using cloth-backed adhesive tape, and the coefficient of friction between the uncoated ripstop fabric and the 3D printed winch material was experimentally measured to be 27.2 kg and 0.2, respectively. The base winch angle limit was set such that the vine robot inner material was wrapped by at least 15.7 radians (250 mm excess length needed for 31.8 mm diameter winch), yielding a load capacity of 629 kg. Thus, if the fastening strength to the base was the bottleneck for the load capacity of the system, given that the base and tip fastenings only bear half of the load each, an object weighing up to 1259 kg could be lifted. This estimate is conservative because it does not account for the increased holding force due to the outer layers applying pressure onto the inner layers, previously demonstrated in *(80)*. In future iterations, the vine robot inner material would be taped directly to the steel shaft without the 3D printed cylinder to reduce the moment arm from the tensile loads, thus reducing the torque on the winch. The shaft is driven by a Neo 550 brushless DC motor (Rev Robotics LLC, TX, USA) with a 100:1 ratio gearbox (AndyMark Inc., IN, USA) capable of providing a total peak stall torque of 97 Nm. The base of the vine robot's outer material is fit over the outside of the 3D printed collar and fastened using a steel hose clamp and 3D printed clamp inserts.

The pulling strength of both the base and the tip-fastening winch is dependent on the gear ratio of their transmission and the maximum radius of their winch, including the thickness of the

wound material. The retraction mechanisms in both devices are just the winch winding up the length of the vine robot. Thus, for a given actuator with a known stall torque, the lifting capacity is dependent only on the torque amplification of the transmission and the moment arm of the winch (i.e. force = torque / radius). The base and tip-fastening winch use the same motor with a stall torque of 0.97 Nm, and have transmission ratios of 100:1 and 200:1, respectively. The winch radii of the base and tip devices before any length of the vine robot has been retracted are 15.9 mm and 63.5 mm, respectively. Thus, the maximum lifting force capacities for the base and tip devices are 623 kg and 311 kg, enabling an object weighting up to 1246 kg and 622 kg to be lifted, respectively. Given an infinite available vine robot length, the maximum radii that the base and tip devices can accommodate due to the added thickness of the vine robot wrapping around the winch are 60.3 mm and 96.3 mm, respectively. Thus, at its maximum retraction length, the lifting force capacities for the base and tip devices are 164 kg and 205 kg, enabling an object weighing up to 328 kg and 410 kg to be lifted, respectively.

Our implementation of the tip-fastening mechanism is a winch with an embedded clamping mechanism to clamp the tip of the vine robot and wind it up to pull on the payload, as illustrated in Fig. 3B. The body of the winch is constructed primarily from 3D printed parts, aluminum plates, and a 15.9 mm wide steel hex shaft, with 9.5 - 12.7 mm (0.375 - 0.5 in) diameter partially-threaded bolts used to reinforce the 3D printed parts in addition to fastening them together. The winch is also driven by a Neo 550 brushless DC motor with a 100:1 ratio gearbox, with an additional 2:1 sprocket-chain transmission. The embedded clamp mechanism utilizes the winch body as the jaws to maintain a compact body, in that a section of the winch can be separated from the main body to open up the clamp. When the clamp is closed, the cylindrical shape of the winch is restored. The clamp is opened and closed by two lead screw linear actuators (Eco-Worthy, China) with a 1500 N pulling capacity, respectively. The non-backdrivable nature allows the high clamping force to be passively maintained. The surfaces of the jaws were designed to maximize the friction on the vine robot by (1) lining them with a nonslip film (Dycem, RI, USA), and (2) implementing an interlocking wave surface pattern into the surfaces of the jaws. As discussed in "Component design" section, clamping the vine robot between the wave-patterned jaws wraps it around a series of curves (with equal radius and arc length) that amplifies the load capacity relative to the holding force (frictional clamping force without the wave pattern) through the capstan friction effect. The clamp was designed with n=8 equivalent curve segments with $\theta_c = \frac{pi}{2}$ (n reduced from actual value of n = 9 to account for fillets reducing θ_c for the curves at the ends of the chain), and two non-backdrivable linear actuators that apply a combined clamping force of F_{clamp} = 299.4 kg. The critical coefficient of friction is between the vine robot inner material and the inside of its outer material. This is because load is borne by the inner material of the vine robot when its length is retracted by the base (inner material shortens, outer materials becomes slack), meaning the inner material can slip through the outer material before the outer material slips relative to the clamp if the friction inside the vine robot is lower than that between the outer material and the jaws. The inside of the vine robot is not coated with TPU while the outside is, and thus the coefficient of friction between the inner material and the inside of the outer material is lower than that between the outer material and the jaws. The inner material coefficient of friction (i.e., non-coated ripstop fabric with itself) was experimentally measured to be 0.20. Thus, given equation S9 and the equation for closed-loop load capacity $F_{load} = 2T_{l,n}$, the theoretical load capacity is 2021 kg.

The pressure in the base is commanded using a QB4TANKKZP25PSG Electro-Pneumatic Pressure Regulator (Proportion-Air, Inc. IN, USA). All electronics are powered using a 110V AC to 12V DC AC-to-DC Power Converter (NUOFUWEITM Guangdong, China).

For the “grasping heavy yet fragile objects” demonstration in which a watermelon was grasped and lifted, a different, smaller grasping device designed within the same system architecture was used. The bounding boxes of its bases are 4.35% the size of the bases used in the human lifting demonstration by volume ($6.28 \times 10^{-4} m^3$ vs. $1.44 \times 10^{-2} m^3$), and the diameters of its vine robots are 32.9% of those used in the human lifting demonstration (25 mm vs. 76 mm). Its bases and tip-fastening mechanisms are made primarily out of 3D printed Polylactic acid (PLA) instead of aluminum, and its vine robots are made from low-density polyethylene (LDPE) instead of nylon fabric, which has a lower but still sufficient tensile strength of 10 MPa *(77)*.

For the demonstration of grasping a ball in a woven configuration, the designs of the vine robot, base, and tip-fastening mechanism were the same designs used in the watermelon grasping and lifting demonstration. Instead of using two vine robots parallel to each other, this system consisted of four 24.3 mm diameter vine robots that grow downward to the side of the object and then bend to grow under the object in a woven warp-weft pattern, each in a direction offset 90° from the last, as shown in Fig.4A.

For the demonstration of the topologically interlocking grasp with the bucket handle (final clip in Movie S2), the large scale system was used. In the “Grasp Versatility” section, we state that this interlocking grasp is theoretically infinitely stable, assuming no breaks in the loops. Notably, to uphold this assumption, the strength of the system can theoretically be scaled without affecting the grasp. For the connections with the ground, the fastening mechanism can be made theoretically infinitely strong, since the fastening strength does not impact the interaction between the grasping mechanism and the object. For the base connection, high-strength fastening is made trivial through the use of standard fastener hardware and/or adhesives. For the tip-fastening mechanism, high-strength holds can be achieved either with high-strength actuators, or better yet, with latching and clutching mechanisms that can powerfully hold the mechanism without needing powerful actuators *(80)*. Thus, assuming the base and tip grounding connections are sufficiently strong, the bottleneck for the grasping device’s pull-out force becomes the tensile yield force of the closed-loop mechanism, which can be made extremely high using high-strength fabrics such as nylon ripstop and/or reinforcing the structure with high-strength fibers such as Kevlar and carbon fiber threads/filaments.

S7. Derivation of tip-fastening winch mechanism load capacity model

The wave-patterned jaws and the belt (i.e. the deflated vine robot membrane) clamped between them are illustrated in Fig. S5. θ_i is the arc length of curve i, n is the number of curves in sequence, $T_{hold,i} = T_{h,i}$ and $T_{load,i} = T_{l,i}$ are the initial and amplified tensile load capacities of the belt before and after being wrapped around curve i, respectively, and μ is the coefficient of friction between the jaws and the belt material. The bend angle that the belt experiences when passing onto the first curve from the previous adjacent flat segment of the jaws, and when passing from the last curve onto its adjacent flat segment, are denoted as ϕ_h and ϕ_l, respectively.

Based on these definitions and given the Euler-Eytelwein formula: $T_l = T_h e^{\mu\theta}$, the holding and loading forces of each adjacent curve pair can be expressed as:

$$T_{h,i+1} = T_{l,i} = T_{h,i}e^{\mu\theta_i} \tag{S6}$$

The amplified tensile load capacity of a curve *i* can then be expressed in terms of the holding force of the initial curve as:

$$T_{l,i} = T_{h,i}e^{\mu\theta_i} = T_{l,i-1}e^{\mu\theta_i} = T_{h,i-1}e^{\mu\theta_{i-1}}e^{\mu\theta_i} = T_{l,i-2}e^{\mu\theta_{i-1}}e^{\mu\theta_i} \tag{S7}$$
$$\ldots = T_{h,1}e^{\mu\theta_1}\ldots e^{\mu\theta_{i-1}}e^{\mu\theta_i} = T_{h,1}e^{\mu\Sigma_1^i\theta_i}$$

To include the amplifications of all of the curves as well as the bends in the belts at the transitions onto the flat segments at the ends of the chain of curves, as well as estimate the tensile holding force of the first curve as the unamplified frictional holding force from the clamping force $T_{h,1} = \mu F_{clamp}$, equation 2 can be expressed as:

$$T_{l,n} = T_{h,1}e^{\mu(\phi_h+\Sigma_1^n\theta_i+\phi_l)} = \mu F_{clamp}e^{\mu(\phi_h+\Sigma_1^n\theta_i+\phi_l)} \tag{S8}$$

In the case of our tip-fastening winch design, $\theta_i = \theta_c$ for all i, and $\phi_h = \phi_l = \frac{1}{2}\theta_c$. Thus,

$$T_{l,n} = \mu F_{clamp}e^{\mu\left(\frac{1}{2}\theta_c+n\theta_c+\frac{1}{2}\theta_c\right)} = \mu F_{clamp}e^{\mu(n+1)\theta_c} \tag{S9}$$

This estimate is also conservative because it does not account for the clamping force that is applied not just at the far end of the chain of curves (where T_{hold} is defined), but also over its entire length.

S8. Robustness of vine robot tip navigation into the tip-fastening mechanism

Building on *(72)*, we generate the path uncertainty of the vine robot as it grows down and under the body, exits from beneath the body and grows into the back wall with tapered ridges. *(72)* establishes how to derive a waypoint and path uncertainty when deploying vine robots to purposefully leverage obstacle collision to reach a target in a known environment. Generating path uncertainty involves sequentially accounting for potential sources of path deviation both in manufacturing the vine and deploying it when deriving the kinematics of the vine robot at each time point. We note our sources of path uncertainty stem from environmental resistance to linear growth during initial entry under the human body, preformed joint manufacturing variability, and a purposefully installed back wall with tapered ridges.

While growing down towards the body, the vine robot encounters no obstacles until it reaches the bed at which we designed a 90 degree preformed joint to pitch towards the human body using fiber reinforced tape to join two points along the vine's growth axis. For a vine robot growing in 3D with preformed joints formed using fiber reinforced tape, *(81)* reports a joint angle uncertainty of approximately +/- 3 degrees. For added robustness, we expand this uncertainty 5-fold in our calculation of path uncertainty to +/- 15 degrees. At both ends of the joint angle range of possible values for this first bend, the vine robot still grows towards the patient.

During initial entry under the body, the robot tip is susceptible to some amount of lateral deflection along the side of the body before generating sufficient tip-driving force to evert under the body. For our system, we observed this to be approximately +/- 20 degrees in practice. Once

everted underneath the body, the vine grows in a straight line, assuming there are no occluding anatomies in its path. However, this lateral deflection induces a tip pivot about the body yaw angle and determines the point along the human body at which the vine robot exits on the side of the human body most proximal to the installed back wall.

At this point, we manufactured a 45° turn along the growth axis to pitch the vine tip up against gravity and towards the target region of within the ridge of the back wall. The back wall with ridges serves as a known environmental object designed to guide the vine robot's vertical growth into the tip clutching winch. Its max width at its interface with the bed is maximized for capturing the full length of the bed. It is parameterized using six spatial reference points, which are projected into the global XZ-plane to construct a two-dimensional polygon representing the cross-section enclosed by the wall ridges. There is a joint manufacturing uncertainty in the tip pitch and roll angles. At both ends of the pitch joint angle range of possible values for this bend, the vine robot still grows towards the back wall. However, the roll angle uncertainty can result in the tip growth missing the target region of the back wall.

Upon intersecting with the back wall, we denote a vine robot path that intersects within the ridges at favorable heading angles, with respect to the ridges, as a "success" path because it biases tip growth towards the tip clutching winch. The authors have previously shown that the heading angle at which the vine robot tip contacts its environment determines its trajectory based on the reaction forces present at the contact point *(80)*. As presented in *(72)*, to generate the path uncertainty of the vine robot shown in Fig. S7, we rely on the vine robot's linear tip-driven growth and preformed joint formations to sequentially plot all the possible paths the robot's tip can take after encountering a known environmental obstacle. Of the paths generated, 92.4% percent result in a favorable path to reach the target.

<u>Pseudocode generating path uncertainty plot (Refer Fig. S7)</u>

--- Define wall ridges geometry ---

Set start point, Define wall ridges corner points (pt1 ... pt6)

Extract X, Z coordinates of funnel relative to start

Create wall ridge polygon (polyshape) in XZ plane

Ridge_plane_depth = bed_length + offset

Define ridge taper vectors (left side, right side) and normalize them

Define ridge plane (point + normal vector)

--- Define bed geometry ---

bed_width = constant, bed_length = constant

--- Define joint uncertainty parameters ---

yaw_angles = range of yaw angles (-15° to +15°)

roll_angles = range of roll angles (-15° to +15°)

--- Initialize storage ---

intersection_points = empty array

is_inside_polyshape = empty logical array

Create homogeneous transform (H_Rx) for body pitch (45°)

--- Loop over yaw angles (outer loop) ---

FOR each yaw_angle:

Create homogeneous transform (H_Rz) for yaw

Define translation (Tx, Ty, Tz) to vine exit point from under obstacle based on yaw

```
Create translation matrix (T)

--- Loop over roll angles (inner loop) ---
FOR each roll_angle:

    Create homogeneous transform (H_Ry) for body roll

    Combine transformations (matching vine growth sequence):
        H_Final    = T * H_Rz * H_Ry  * H_Rx

    Extract transformed vine growth direction

    Project direction onto wall ridge plane
    Normalize projected vector

    --- Heading angle check ---
    IF yaw_angle < 0:
        Compute angle between projection and left-side funnel vector
    ELSE:
        Compute angle between projection and right-side funnel vector
    ENDIF

    Convert angle to degrees
    If angle > 90° → mark path as favorable
    Else → unfavorable

    Compute growth direction line to back wall plane intersection

    IF (No intersection):
        Parallel
    ELSE:
        Solve for intersection point
        Check if intersection lies inside funnel polyshape
        If inside → keep path as favorable
        If outside → mark as unfavorable (entry=0)
    ENDIF

END FOR (roll loop)


END FOR (yaw loop)


--- Calculate success/failure rate ---
```

success_rate = fraction of favorable paths
fail_rate = fraction of unfavorable paths

--- Visualization ---

Initialize 3D plot

Draw global coordinate axes

Draw wall ridge plane and polygon

FOR each yaw/roll iteration:

    Recompute transformation
    Plot vine growth paths
    Plot intersection points

END FOR

S9. Human grasping and lifting demonstration – participant interview responses

Each of the following questions were verbally asked to the subject in person after they complete all experimental procedures.

Question 1: Please rate the level of pain you experienced while being lifted by the robotic harness system on a scale of 1 to 7 (1 = none, 7 = unbearable).

Response 1: 1 (“No pain.”)

Question 2: Please rate the level of discomfort you experienced while being lifted by the robotic harness system on a scale of 1 to 7 (1 = none, 7 = unbearable).

Response 2: 3 (“Holding my head up while the harness was lifting, produced a bit of strain on my neck and head.

S10. Human contact pressure experiment

We measured the pressure distribution on the lower back of a 79.4kg (175lb) life-sized manikin (Simulaids I.A.F.F. Rescue Randy) lying in a supine position on a bed while a vine robot grows between its back and the bed to harness it. During the experiment, the vine robot grows downward out of its base until it reaches the mattress, at which point it is actuated to grow along the surface of the mattress toward and then underneath the manikin body without sliding friction.

The manikin was outfitted with a custom soft capacitive sensor array created by Pressure Profile Systems (Los Angeles, CA, US) on its lower back as shown in Fig 5. The sensor array records pressure data in 6.45 x 10-4 m^2 cells at 20 Hz. The device has a range from 0 kPa to 20.48 kPa with 0.0276 ± 0.00689kPa measured resolution. It was calibrated linearity of 99.9 ± 0.1% and signal-to-noise ratio of 732 ± 219. The sensor array covered a total area 0.0387 m^2 (0.38m x 0.1m).

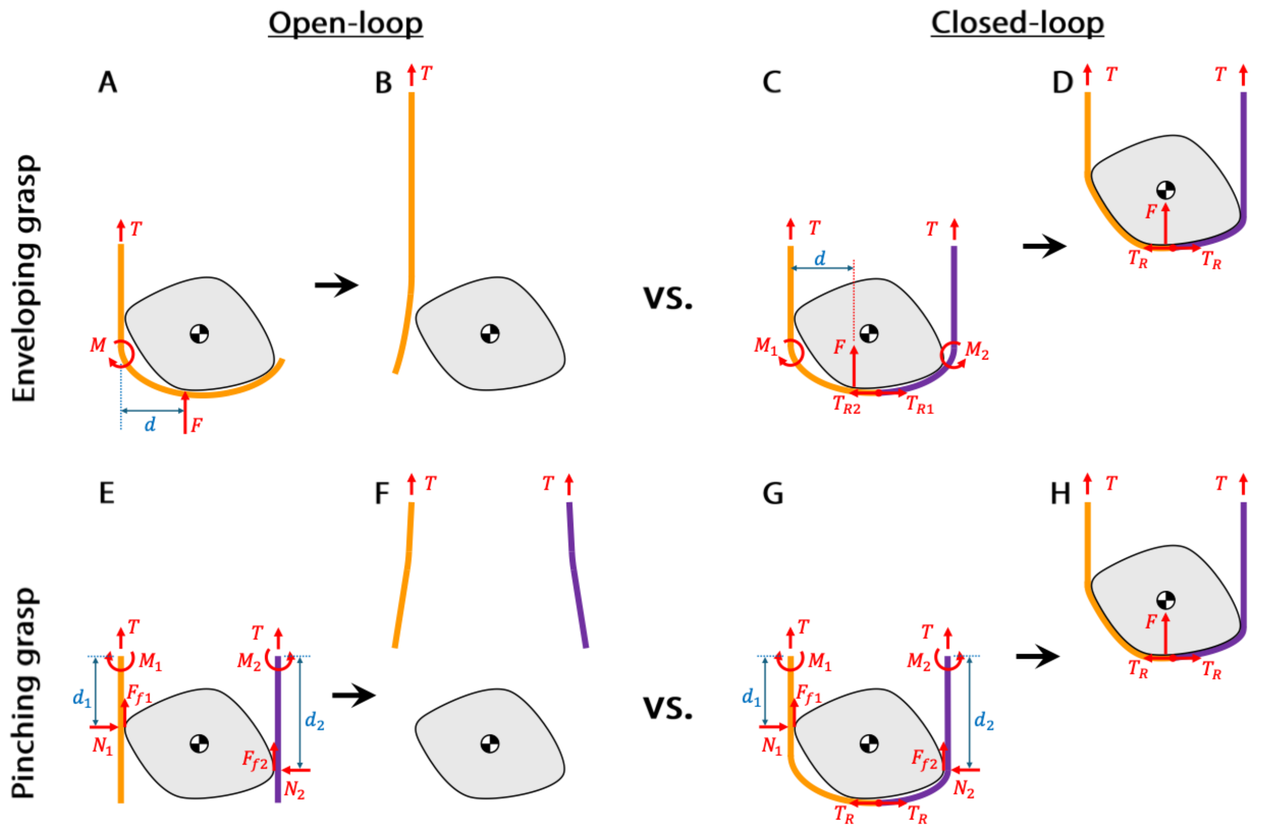

Fig. S1.

Closed-loop vs. open-loop grasping morphologies. (A-H) Force diagrams of open- loop grasping mechanisms before and after pulling on objects for enveloping grasping and pinching grasping, and their associated closed-loop mechanism comparisons. T, M, T_R, F, F_f, N, and d denote pulling tension, internal bending moment, internal reaction tension, pushing force, frictional force, normal pinching force, and moment arm, respectively.

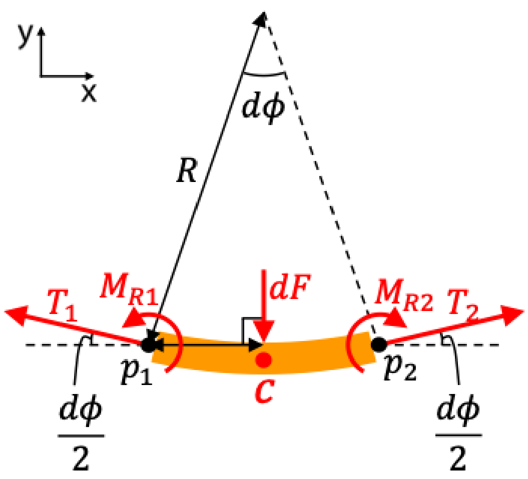

Fig. S2.

Free body diagram for a differential segment of a grasping mechanism. Differential segment associated with contact point c.

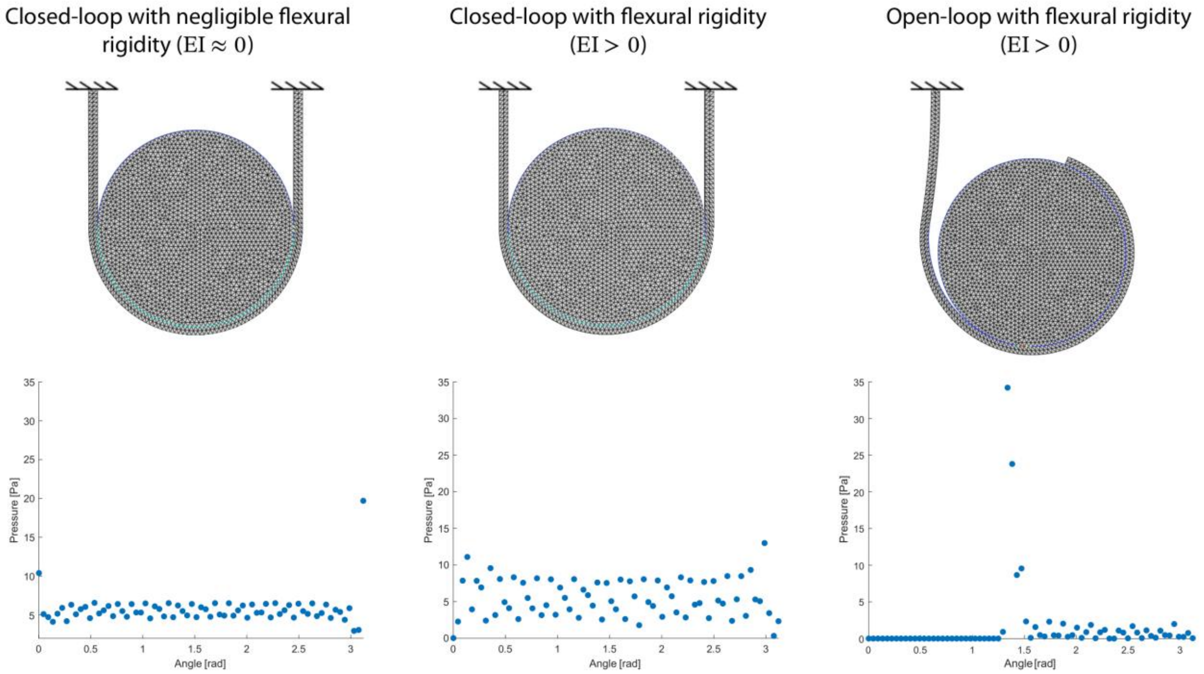

Fig. S3.

Closed-loop vs. open-loop mechanism pressure distribution finite element analysis.
Comparison of contact pressure profiles for an object grasped by a closed-loop mechanism with elastic modulus $E \approx 0$ (left), a closed-loop mechanism with E=125 MPa (center), and an open-loop with E= 125 MPa (right).

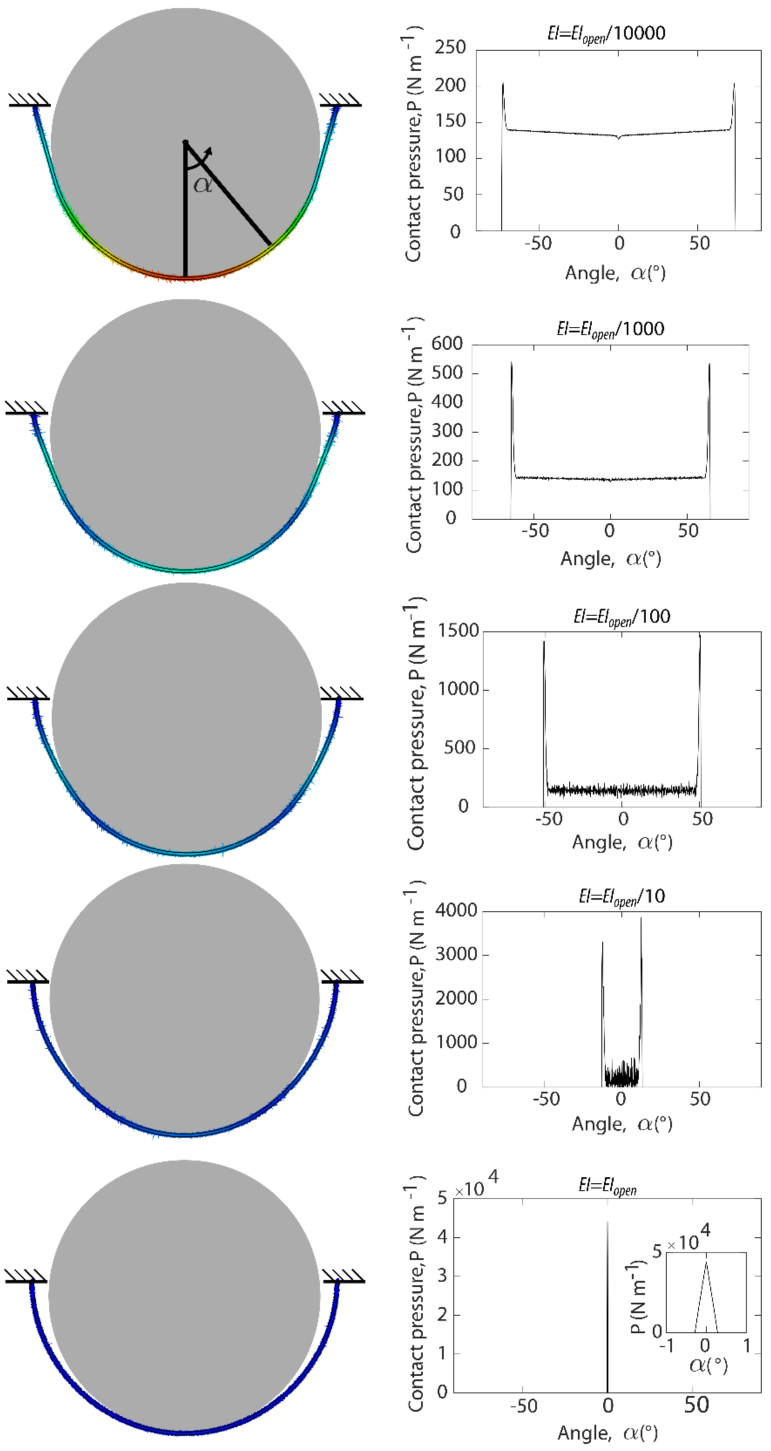

Fig. S4.

Pressure distributions between a circular object and five different closed-loop mechanisms with different flexural rigidities. α is the angle of wrapping of the loop around the object, with $\alpha = 0$ directly below the geometric center of the object. As flexural rigidity increases, the contact pressure concentrations at the touch-down and lift-off points become greater, increasing the maximum pressure on the object.

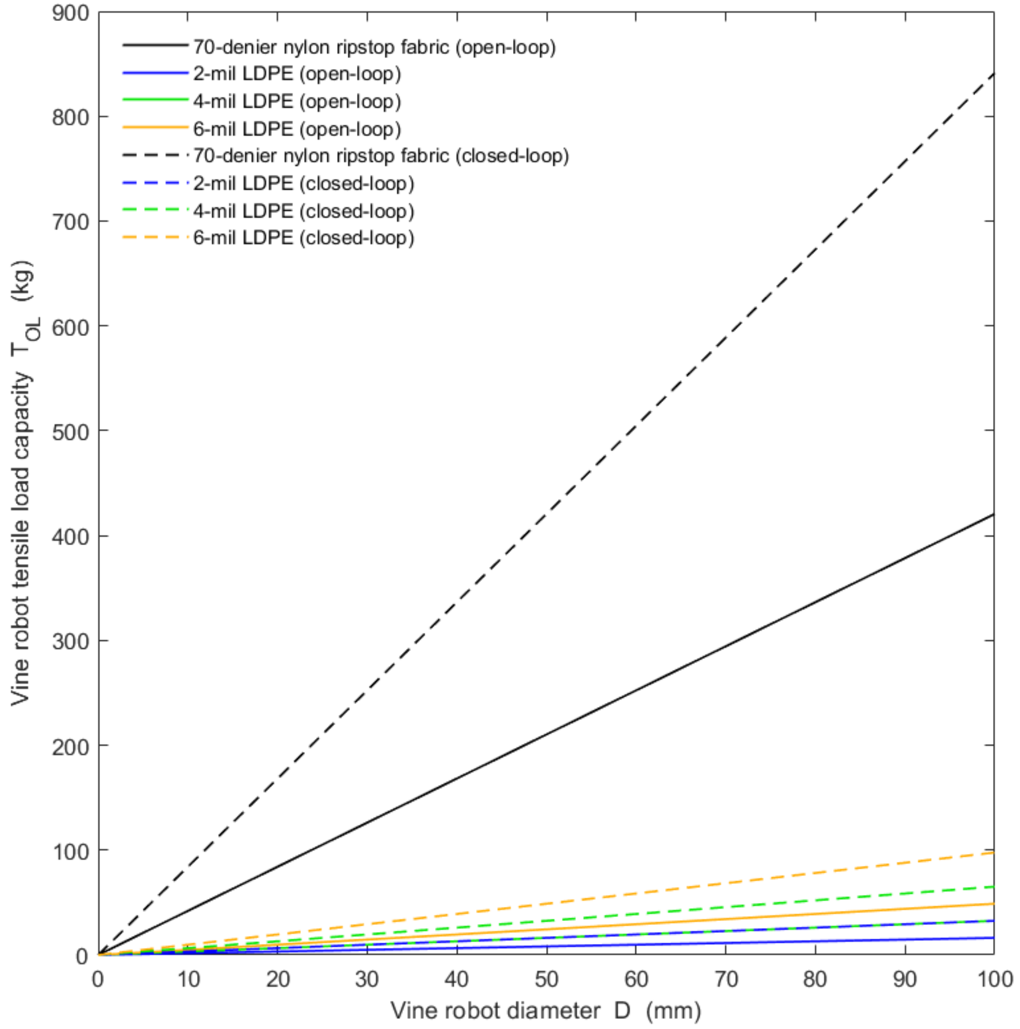

Fig. S5.

The scaling relationships between the tensile load capacity of vine robots with respect to their diameter for different membrane materials. The theoretical load capacity of the vine robot in the closed-loop configuration is twice that of the open-loop configuration.

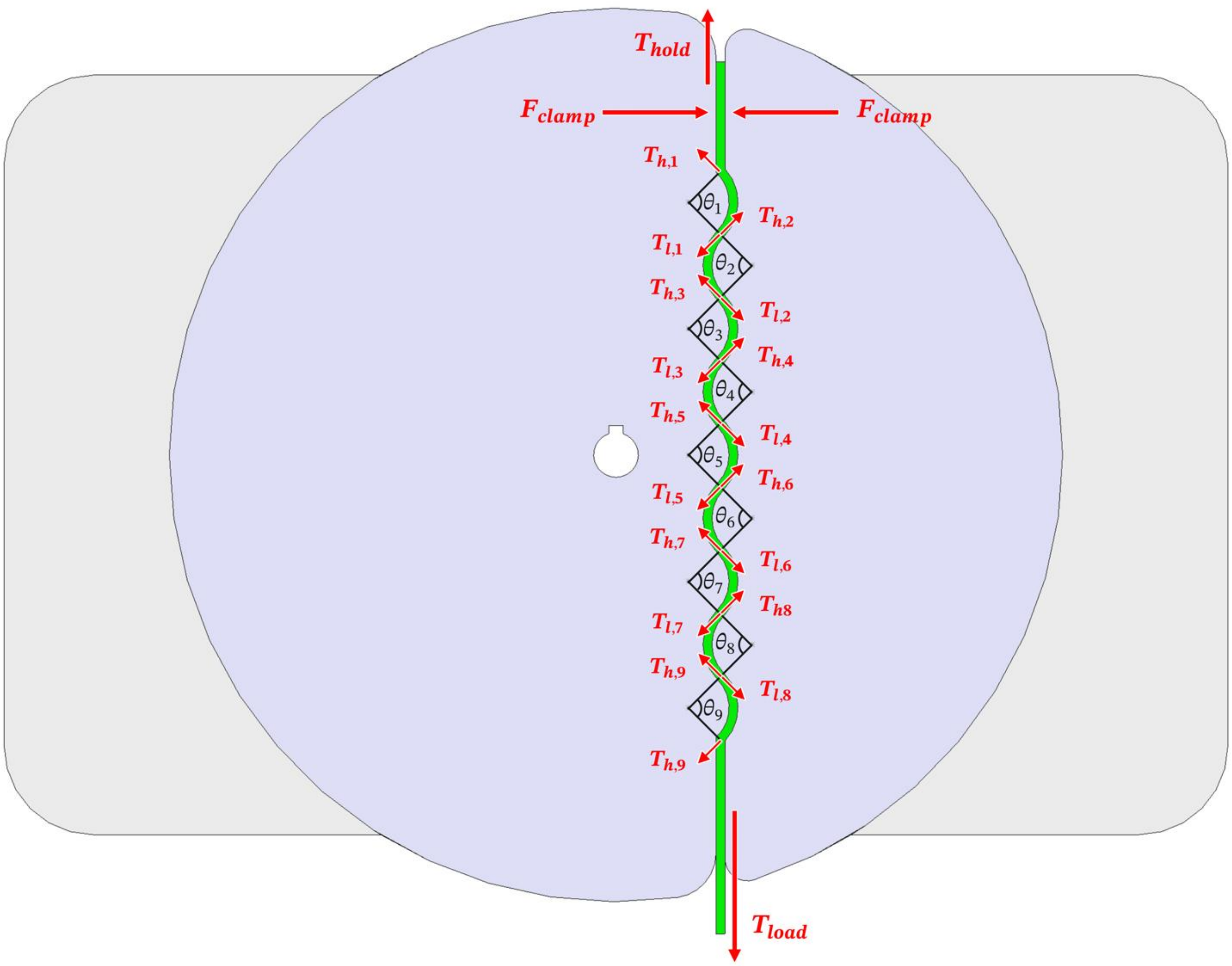

Fig. S6.

Wave pattern of jaws clamping deflated vine robot in tip-fastening winch mechanism.
Capstan friction between vine robot and curved segments of clamp jaws amplifies the clamping force to yield a high load capacity.

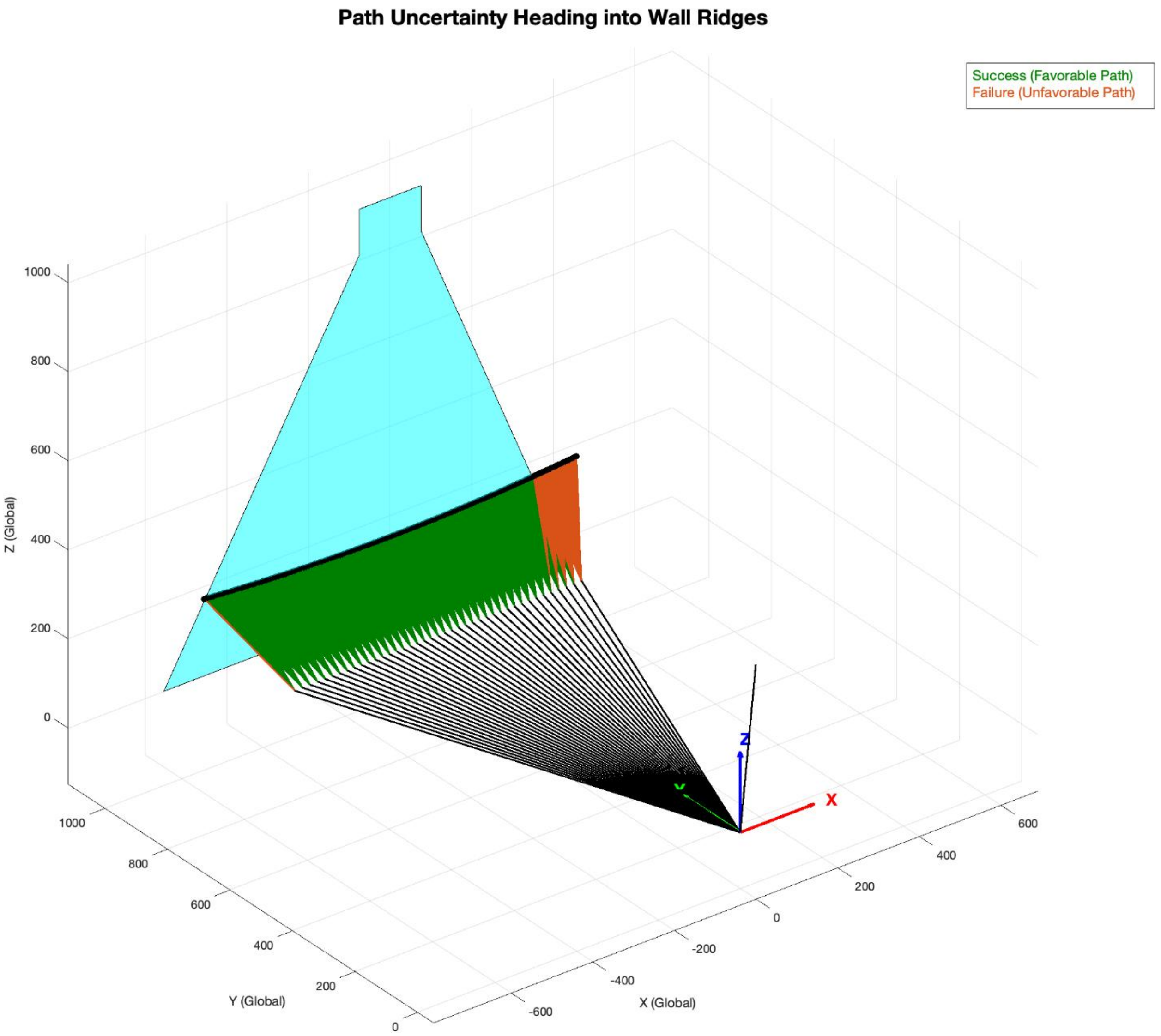

Fig. S7.
Vine robot tip growth path uncertainty for human lift demonstrations.

Description of Movies

Movie S1: Woven grasp configuration. Small-scale loop closure grasping system grasps, lifts, and releases a ball with the linkages creating a woven caging configuration.

Movie S2: Grasping via interlocking closed loops. Small-scale loop closure grasping system creates and releases interlocked Hopf link grasps with a ring, and large-scale loop closure grasping system creates and releases interlocked Hopf link grasps with a bucket handle.

Movie S3: Grasping in a cluttered environment. Small-scale loop closure grasping system grasps and lifts a kettle bell weight from inside a cluttered bin filled with plastic objects. The soft growing linkage mechanism grows through spaces smaller than its diameter to navigate through the objects into the desired grasping configuration.

Movie S4: Antagonistic insertion of linkages between a vase and its resting surface. Small-scale loop closure grasping system grasping and lifting a glass vase from its resting surface with no preexisting gap. Three soft growing linkage mechanisms grow through the interface between the vase and table from opposite directions to balance the horizontal pushing force that each exerts on the object, and navigate past the interface into the desired grasping configuration.

Movie S5: Long distance grasp. Large-scale loop closure grasping system grasps and pulls boxes from 3 m away, leveraging the far-reaching deployability of vine robots from compact bases.

Movie S6: Simultaneous multi-object grasping. Small-scale loop closure grasping system grasps, transfers, and releases a pile of eight pipes in an single bundle.

Movie S7: "In-hand" manipulation. Small-scale loop closure grasping system grasps, lifts, and rolls a cylinder in place mid-air by retracting and extending the two ends of the loops at equal and opposite speeds.

Movie S8: Grasping a human lying on a bed. Large-scale loop closure grasping system grasps, lifts, and releases a human subject lying on a bed.

Movie S9: Eversion pressure under life-sized manikin experiment. Contact pressure map over time between a life-sized weighted manikin and a soft growing linkage mechanism of the large-scale system as it grows under their body.

Movie S10: Grasping and lifting a watermelon. Small-scale loop closure grasping system grasps, transfers, and releases a 5.9 kg watermelon.

Description of Data

Data S1. (separate file)
Raw data for finite element analysis results shown in Fig. S4.

Data S2. (separate file)
Raw data for finite element analysis results shown in Fig. S3.

Data S3. (separate file)
Raw data for human harnessing contact pressure measurement experiment (shown in Fig.6).

REFERENCES AND NOTES

1. Z. Samadikhoshkho, K. Zareinia, F. Janabi-Sharifi, "A brief review on robotic grippers classifications," in *2019 IEEE Canadian Conference of Electrical and Computer Engineering (CCECE)* (IEEE, 2019), pp. 1–4.

2. J. Shintake, V. Cacucciolo, D. Floreano, H. Shea, Soft robotic grippers. *Adv. Mater.* **30**, 1707035 (2018).

3. J. Hughes, U. Culha, F. Giardina, F. Guenther, A. Rosendo, F. Iida, Soft manipulators and grippers: A review. *Front. Robot. AI* **3**, 69 (2016).

4. K. Tai, A.-R. El-Sayed, M. Shahriari, M. Biglarbegian, S. Mahmud, State of the art robotic grippers and applications. *Robotics* **5**, 11 (2016).

5. C. Piazza, G. Grioli, M. G. Catalano, A. Bicchi, A century of robotic hands. *Annu. Rev. Control Robot. Auton. Syst.* **2**, 1–32 (2019).

6. A. Billard, D. Kragic, Trends and challenges in robot manipulation. *Science* **364**, eaat8414 (2019).

7. S. C. deWit, P. A. Williams, *Fundamental Concepts and Skills for Nursing* (Elsevier, 2013).

8. Z. Feng, Global convergence: Aging and long-term care policy challenges in the developing world. *J. Aging Soc. Policy* **31**, 291–297 (2019).

9. S. A. Lavender, K. M. Conrad, P. A. Reichelt, A. K. Kohok, J. Gacki-Smith, Designing ergonomic interventions for emergency medical services workers—Part III: Bed to stairchair transfers. *Appl. Ergon.* **38**, 581–589 (2007).

10. A. E. Fürst, R. Keller, M. Kummer, C. Manera, B. Von Salis, J. Auer, R. Bettschart-Wolfensberger, Evaluation of a new full-body animal rescue and transportation sling in horses: 181 horses (1998–2006). *J. Vet. Emerg. Crit. Care* **18**, 619–625 (2008).

11. M. Samdal, H. Eiding, L. Markengbakken, J. Røislien, M. Rehn, M. Sandberg, Time course of hoist operations by the search and rescue helicopter service in Southeast Norway. *Wilderness Environ. Med.* **30**, 351–361 (2019).

12. D. Penrose, *Occupational Therapy for Orthopaedic Conditions* (Springer, 1993).

13. K. E. Norman, A. Pepin, M. Ladouceur, H. Barbeau, A treadmill apparatus and harness support for evaluation and rehabilitation of gait. *Arch. Phys. Med. Rehabil.* **76**, 772–778 (1995).

14. H. Srivatsan, A. V. Myagerimath, V. G. Duffy, "A systematic review of collaborative robots in ergonomics," in *Digital Human Modeling and Applications in Health, Safety, Ergonomics and Risk Management* (Springer, 2024), pp. 282–297.

15. R. Bostelman, J. Albus, N. Dagalakis, A. Jacoff, "RoboCrane project: An advanced concept for large scale manufacturing," in *Proceedings of the AUVSI Conference*, Orlando, FL, 1 July 1996 (AUVSI, 1996).

16. R. Hoffman, H. H. Asada, Precision assembly of heavy objects suspended with multiple cables from a crane. *IEEE Robot. Autom. Lett.* **5**, 6876–6883 (2020).

17. J. O. Glerum, S. Kelly, *Stage Rigging Handbook* (Southern Illinois Univ. Press, ed. 3, 2007).

18. B. Kelechava, "ASME B30.9-2021: Slings," *ANSI Blog*, 20 January 2022; https://blog.ansi.org/ansi/asme-b30-9-2021-slings/.

19. D. Ibekwe, "It took 2 cranes to lift the 41-tonne plane that skidded off an icy runway in Turkey," *Business Insider*, 20 January 2018; www.businessinsider.com/2-cranes-recover-plane-skidded-off-runway-turkey-trabzon-airport-2018-1.

20. Crane Lifting Slings, "Bridles and assemblies," *Tri-State Rigging Equipment*; https://tsriggingequipment.com/crane-lifting-slings-bridles-assemblies.

21. B. Roy, A. Basmajian, H. Asada, “Maneuvering a bed sheet for repositioning a bedridden patient,” in *2003 IEEE International Conference on Robotics and Automation (ICRA)* (IEEE, 2003), pp. 2224–2229.

22. M. Mooney, “BMW scales up use of 3D-printed robot grippers for car assembly,” *Robotics and Automation*, 24 May 2024; www.roboticsandautomationmagazine.co.uk/news/assembly/bmw-scales-up-use-of-3d-printed-robot-grippers-for-car-assembly.html.

23. E. E. Phillips, “Massive robots keep docks shipshape,” *Wall Street* Journal, 27 March 2016; www.wsj.com/articles/massive-robots-keep-docks-shipshape-1459104327.

24. T. Mukai, S. Hirano, H. Nakashima, Y. Kato, Y. Sakaida, S. Guo, S. Hosoe, “Development of a nursing-care assistant robot RIBA that can lift a human in its arms,” in *2010 IEEE/RSJ International Conference on Intelligent Robots and Systems (IROS)* (IEEE, 2010), pp. 5996–6001.

25. C. Loh, H. Tsukagoshi, “Pneumatic Big-hand gripper with slip-in tip aimed for the transfer support of the human body,” in *2014 IEEE International Conference on Robotics and Automation (ICRA)* (IEEE, 2014), pp. 475–481.

26. R. Baines, S. K. Patiballa, J. Booth, L. Ramirez, T. Sipple, A. Garcia, F. Fish, R. Kramer-Bottiglio, Multi-environment robotic transitions through adaptive morphogenesis. *Nature* **610**, 283–289 (2022).

27. E. Sihite, A. Kalantari, R. Nemovi, A. Ramezani, M. Gharib, Multi-modal mobility morphobot (M4) with appendage repurposing for locomotion plasticity enhancement. *Nat. Commun.* **14**, 3323 (2023).

28. J. Sun, E. Lerner, B. Tighe, C. Middlemist, J. Zhao, Embedded shape morphing for morphologically adaptive robots. *Nat. Commun.* **14**, 6023 (2023).

29. T. F. Nygaard, C. P. Martin, J. Torresen, K. Glette, D. Howard, Real-world embodied AI through a morphologically adaptive quadruped robot. *Nat. Mach. Intell.* **3**, 410–419 (2021).

30. T. J. Cairnes, C. J. Ford, E. Psomopoulou, N. Lepora, An overview of robotic grippers. *IEEE Potentials* **42**, 17–23 (2023).

31. J. Hernandez, M. S. H. Sunny, J. Sanjuan, I. Rulik, M. I. I. Zarif, S. I. Ahamed, H. U. Ahmed, M. H. Rahman, Current designs of robotic arm grippers: A comprehensive systematic review. *Robotics* **12**, 5 (2023).

32. D. Prattichizzo, J. C. Trinkle, Grasping, in *Springer Handbook of Robotics* (Springer, 2016), pp. 955–988.

33. K. Becker, C. Teeple, N. Charles, Y. Jung, D. Baum, J. C. Weaver, L. Mahadevan, R. Wood, Active entanglement enables stochastic, topological grasping. *Proc. Natl. Acad. Sci. U.S.A.* **119**, e2209819119 (2022).

34. E. Brown, N. Rodenberg, J. Amend, A. Mozeika, E. Steltz, M. R. Zakin, H. Lipson, H. M. Jaeger, Universal robotic gripper based on the jamming of granular material. *Proc. Natl. Acad. Sci. U.S.A.* **107**, 18809–18814 (2010).

35. F. Aljaber, A. Hassan, T. Abrar, I. Vitanov, K. Althoefer, "Soft inflatable fingers: An overview of design, prototyping and sensorisation for various applications," in *2023 IEEE International Conference on Soft Robotics (RoboSoft)* (IEEE, 2023), pp. 1–6.

36. J. Shintake, S. Rosset, B. Schubert, D. Floreano, H. Shea, Versatile soft grippers with intrinsic electroadhesion based on multifunctional polymer actuators. *Adv. Mater.* **28**, 231–238 (2016).

37. J. Qu, Z. Yu, W. Tang, Y. Xu, B. Mao, K. Zhou, Advanced technologies and applications of robotic soft grippers. *Adv. Mater. Technol.* **9**, 2301004 (2024).

38. G. He, C. Sparks, N. Gravish, Grasping and rolling in-plane manipulation using deployable tape spring appendages. *Sci. Adv.* **11**, eadt5905 (2025).

39. S. Li, D. M. Vogt, D. Rus, R. J. Wood, Fluid-driven origami-inspired artificial muscles. *Proc. Natl. Acad. Sci. U.S.A.* **114**, 13132–13137 (2017).

40. V. Cacucciolo, J. Shintake, H. Shea, "Delicate yet strong: Characterizing the electro-adhesion lifting force with a soft gripper," in *2019 2nd IEEE International Conference on Soft Robotics (RoboSoft)* (IEEE, 2019), pp. 108–113.

41. C. Majidi, Soft robotics: A perspective—Current trends and prospects for the future. *Soft Robot.* **1**, 5–11 (2014).

42. C. Laschi, B. Mazzolai, M. Cianchetti, Soft robotics: Technologies and systems pushing the boundaries of robot abilities. *Sci. Robot.* **1**, eaah3690 (2016).

43. M. Cianchetti, C. Laschi, A. Menciassi, P. Dario, Biomedical applications of soft robotics. *Nat. Rev. Mater.* **3**, 143–153 (2018).

44. O. Yasa, Y. Toshimitsu, M. Y. Michelis, L. S. Jones, M. Filippi, T. Buchner, R. K. Katzschmann, An overview of soft robotics. *Annu. Rev. Control Robot. Auton. Syst.* **6**, 1–29 (2023).

45. A. Fox, "New research suggests humans invented string at least 120,000 years," *Smithsonian Magazine*, 10 July 2020; www.smithsonianmag.com/smart-news/study-suggests-humans-invented-string-least-120000-years-ago-180975286/.

46. The Editors of Encyclopaedia Britannica, Ed., "sling," *Encyclopedia Britannica*, 29 May 2013; www.britannica.com/technology/sling.

47. M. C. Langley, T. Suddendorf, Mobile containers in human cognitive evolution studies: Understudied and underrepresented. *Evol. Anthropol.* **29**, 299–309 (2020).

48. T. Suddendorf, K. Kirkland, A. Bulley, J. Redshaw, M. C. Langley, It's in the bag: Mobile containers in human evolution and child development. *Evol. Hum. Sci.* **2**, e48 (2020).

49. "Hoisting and rigging fundamentals for riggers and operators," (TR244C, Rev. 5, US Department of Energy, 2002); www.energy.gov/sites/prod/files/2014/01/f6/HoistingRigging_Fundamentals.pdf.

50. G. Kang, Y.-J. Kim, S.-J. Lee, S. K. Kim, D.-Y. Lee, K. Song, Grasping through dynamic weaving with entangled closed loops. *Nat. Commun.* **14**, 4633 (2023).

51. L. Manes, S. Fichera, H. Fakhruldeen, A. I. Cooper, P. Paoletti, A soft cable loop based gripper for robotic automation of chemistry. *Sci. Rep.* **14**, 8899 (2024).

52. K. Barhydt, H. H. Asada, A high-strength, Highly-flexible robotic strap for harnessing, lifting, and transferring humans. *IEEE Robot. Autom. Lett.* **8**, 2110–2117 (2023).

53. E. W. Hawkes, L. H. Blumenschein, J. D. Greer, A. M. Okamura, A soft robot that navigates its environment through growth. *Sci. Robot.* **2**, eaan3028 (2017).

54. L. H. Blumenschein, M. M. Coad, D. A. Haggerty, A. M. Okamura, E. W. Hawkes, Design, modeling, control, and application of everting vine robots. *Front. Robot. AI* **7**, 548266 (2020).

55. M. Russo, S. M. H. Sadati, X. Dong, A. Mohammad, I. D. Walker, C. Bergeles, K. Xu, D. A. Axinte, Continuum robots: An overview. *Adv. Intell. Syst.* **5**, 2200367 (2023).

56. X. Dong, D. Axinte, D. Palmer, S. Cobos, M. Raffles, A. Rabani, J. Kell, Development of a slender continuum robotic system for on-wing inspection/repair of gas turbine engines. *Robot. Comput. Integr. Manuf.* **44**, 218–229 (2017).

57. J. Burgner-Kahrs, D. C. Rucker, H. Choset, Continuum robots for medical applications: A survey. *IEEE Trans. Robot.* **31**, 1261–1280 (2015).

58. E. Del Dottore, A. Mondini, N. Rowe, B. Mazzolai, A growing soft robot with climbing plant–inspired adaptive behaviors for navigation in unstructured environments. *Sci. Robot.* **9**, eadi5908 (2024).

59. P. Grandgeorge, T. G. Sano, P. M. Reis, An elastic rod in frictional contact with a rigid cylinder. *J. Mech. Phys. Solids* **164**, 104885 (2022).

60. S. Makita, Y. Maeda, “3D multifingered caging: Basic formulation and planning,” in *2008 IEEE/RSJ International Conference on Intelligent Robots and Systems (IROS)* (IEEE, 2008), pp. 2697–2702.

61. D. Rolfsen, *Knots and Links* (American Mathematical Society, 2003).

62. T. Nakamura, H. Tsukagoshi, “Soft pneumatic manipulator capable of sliding under the human body and its application to preventing bedsores,” in *2018 IEEE/ASME International Conference on Advanced Intelligent Mechatronics (AIM)* (IEEE, 2018), pp. 956–961.

63. L. H. Blumenschein, N. S. Usevitch, B. H. Do, E. W. Hawkes, A. M. Okamura, “Helical actuation on a soft inflated robot body,” in *2018 IEEE International Conference on Soft Robotics (RoboSoft)* (IEEE, 2018), pp. 245–252.

64. M. M. Coad, L. H. Blumenschein, S. Cutler, J. A. R. Zepeda, N. D. Naclerio, H. El-Hussieny, U. Mehmood, J.-H. Ryu, E. W. Hawkes, A. M. Okamura, Vine robots. *IEEE Robot. Autom. Mag.* **27**, 120–132 (2020).

65. E. W. Hawkes, C. Xiao, R.-A. Peloquin, C. Keeley, M. R. Begley, M. T. Pope, G. Niemeyer, Engineered jumpers overcome biological limits via work multiplication. *Nature* **604**, 657–661 (2022).

66. S.-G. Jeong, M. M. Coad, L. H. Blumenschein, M. Luo, U. Mehmood, J. H. Kim, A. M. Okamura, J.-H. Ryu, “A tip mount for transporting sensors and tools using soft growing robots,” in *2020 IEEE/RSJ International Conference on Intelligent Robots and Systems (IROS)* (IEEE, 2020), pp. 8781–8788.

67. A. M. Kübler, S. U. Rivera, F. B. Raphael, J. Förster, R. Siegwart, A. M. Okamura, “A multi-segment, soft growing robot with selective steering,” in *2023 IEEE International Conference on Soft Robotics (RoboSoft)* (IEEE, 2023), pp. 1–7.

68. R. Jitosho, S. Simón-Trench, A. M. Okamura, B. H. Do, “Passive shape locking for multibend growing inflated beam robots,” in *2023 IEEE International Conference on Soft Robotics (RoboSoft)* (IEEE, 2023), pp. 1–6.

69. L. Euler, Remarques sur l’effet du frottement dans l’équilibre. *Mémoires de l’académie des sciences de Berlin* **18**, 265–278 (1769).

70. C. C. Adams, *The Knot Book: An Elementary Introduction to the Mathematical Theory of Knots* (American Mathematical Society, 2004).

71. F. Sherwani, M. M. Asad, B. Ibrahim, “Collaborative robots and industrial revolution 4.0 (IR 4.0),” in *2020 International Conference on Emerging Trends in Smart Technologies* (*ICETST)* (IEEE, 2020), pp. 1–5.

72. J. D. Greer, L. H. Blumenschein, R. Alterovitz, E. W. Hawkes, A. M. Okamura, Robust navigation of a soft growing robot by exploiting contact with the environment. *Int. J. Robot. Res.* **39**, 1724–1738 (2020).

73. M. J. Peterson, J. A. Kahn, M. V. Kerrigan, J. M. Gutmann, J. J. Harrow, Pressure ulcer risk of patient handling sling use. *J. Rehabil. Res. Dev.* **52**, 291–300 (2015).

74. M. Jiang, Q. Yu, N. Gravish, “Vacuum induced tube pinching enables reconfigurable flexure joints with controllable bend axis and stiffness,” in *2021 IEEE 4th International Conference on Soft Robotics (RoboSoft)* (IEEE, 2021), pp. 315–320.

75. M. Wang, X. Dong, W. Ba, A. Mohammad, D. Axinte, A. Norton, Design, modelling and validation of a novel extra slender continuum robot for in-situ inspection and repair in aeroengine. *Robot. Comput. Integr. Manuf.* **67**, 102054 (2021).

76. M. Kaneko, N. Kanayama, T. Tsuji, Active antenna for contact sensing. *IEEE Trans. Rob. Autom.* **14**, 278–291 (1998).

77. O. Szlachetka, J. Witkowska-Dobrev, A. Baryła, M. Dohojda, Low-density polyethylene (LDPE) building films – Tensile properties and surface morphology. *J. Build. Eng.* **44**, 103386 (2021).

78. A. Rodriguez, M. T. Mason, S. Ferry, From caging to grasping. *Int. J. Robot. Res.* **31**, 886–900 (2012).

79. H. Hanafusa, H. Asada, Stable prehension of objects by the robot hand with elastic fingers. *Trans. Soc. Instrum. Control Eng.* **13**, 370–377 (1977).

80. O. G. Osele, K. Barhydt, N. Cerone, A. M. Okamura, H. Harry Asada, “Tip-clutching winch for high tensile force application with soft growing robots,” in *2024 IEEE International Conference on Robotics and Automation (ICRA)* (IEEE, 2024), pp. 9362–9368.

81. N. Agharese, “Modeling and interfacing with vine robots,” thesis, Stanford University, Stanford, CA (2023).
